# Spermatogonial fate in mice with increased activin A bioactivity and testicular somatic cell tumours

**DOI:** 10.3389/fcell.2023.1237273

**Published:** 2023-07-26

**Authors:** Penny A. F. Whiley, Benedict Nathaniel, Peter G. Stanton, Robin M. Hobbs, Kate L. Loveland

**Affiliations:** ^1^ Centre for Reproductive Health, Hudson Institute of Medical Research, Clayton, VIC, Australia; ^2^ Department of Molecular and Translational Sciences, School of Clinical Sciences, Monash University, Clayton, VIC, Australia

**Keywords:** SSC, stem cell, inhibin, spermatogenesis, steroidogenesis

## Abstract

Adult male fertility depends on spermatogonial stem cells (SSCs) which undergo either self-renewal or differentiation in response to microenvironmental signals. Activin A acts on Sertoli and Leydig cells to regulate key aspects of testis development and function throughout life, including steroid production. Recognising that activin A levels are elevated in many pathophysiological conditions, this study investigates effects of this growth factor on the niche that determines spermatogonial fate. Although activin A can promote differentiation of isolated spermatogonia *in vitro*, its impacts on SSC and spermatogonial function *in vivo* are unknown. To assess this, we examined testes of *Inha* KO mice, which feature elevated activin A levels and bioactivity, and develop gonadal stromal cell tumours as adults. The GFRA1+ SSC-enriched population was more abundant and proliferative in *Inha* KO compared to wildtype controls, suggesting that chronic elevation of activin A promotes a niche which supports SSC self-renewal. Intriguingly, clusters of GFRA1+/EOMES+/LIN28A– cells, resembling a primitive SSC subset, were frequently observed in tubules adjacent to tumour regions. Transcriptional analyses of *Inha* KO tumours, tubules adjacent to tumours, and tubules distant from tumour regions revealed disrupted gene expression in each KO group increased in parallel with tumour proximity. Modest transcriptional changes were documented in *Inha* KO tubules with complete spermatogenesis. Importantly, tumours displaying upregulation of activin responsive genes were also enriched for factors that promote SSC self-renewal, including *Gdnf*, *Igf1*, and *Fgf2*, indicating the tumours generate a supportive microenvironment for SSCs. Tumour cells featured some characteristics of adult Sertoli cells but lacked consistent SOX9 expression and exhibited an enhanced steroidogenic phenotype, which could arise from maintenance or acquisition of a fetal cell identity or acquisition of another somatic phenotype. Tumour regions were also heavily infiltrated with endothelial, peritubular myoid and immune cells, which may contribute to adjacent SSC support. Our data show for the first time that chronically elevated activin A affects SSC fate *in vivo*. The discovery that testis stromal tumours in the *Inha* KO mouse create a microenvironment that supports SSC self-renewal but not differentiation offers a strategy for identifying pathways that improve spermatogonial propagation *in vitro*.

## Introduction

Spermatogenesis is the continuous and highly specialised process of cellular differentiation which generates mature spermatozoa. This relies on spermatogonial stem cells (SSCs) in the basal layer of the seminiferous epithelium. SSCs can either self-renew or differentiate to maintain spermatogenesis or rescue it following physiological and environmental insults. In adult mice, SSCs are found within a functionally and molecularly heterogeneous undifferentiated spermatogonial population (type A undifferentiated, A_undiff_ or undiff SPG) (La and Hobbs, 2019). The pool of A_undiff_ spermatogonia consists of single isolated cells (A_s_), pairs of cells (A_pr_), and chains of 4–16 cells (A_al 4–16_) interconnected by cytoplasmic bridges. In homeostatic conditions, SSCs are largely contained within A_s_ and A_pr_ fractions, and the remaining A_undiff_ act as transit-amplifying progenitors primed for differentiation. However, this hierarchical structure is adaptive. Lineage-tracing experiments show that under regenerative conditions, progenitors undergo fragmentation and acquire stem cell potential (Nakagawa et al., 2010), form long GFRA1+ A_al 8–16_ chains, which are rarely observed in steady-state testes, and SSC proliferation increases (La et al., 2018b); the result is replenishment of the SSC pool and restoration of spermatogenesis. Common molecular markers used to identify the self-renewing SSC-enriched population include the receptor GFRA1, which marks the majority of A_s_ and A_pr_, and the transcription factors ID4 (Helsel et al., 2017) and EOMES which mark a primitive subset of GFRA1+ cells (La and Hobbs, 2019; Sharma et al., 2019). Transit-amplifying progenitor markers include LIN28A (Chakraborty et al., 2014) and SOX3 (predominantly in A_al_) (McAninch et al., 2020), while SALL4 and PLZF are expressed throughout the A_undiff_ population and at initial differentiation stages, while KIT identifies differentiating spermatogonia (diff SPG) (Yoshinaga et al., 1991). GILZ is broadly expressed throughout the germline but is predominant in A_undiff_ and early meiotic cells (La et al., 2018a).

SSC cell fate (self-renewal vs differentiation) is influenced by niche-derived growth factors, including glial cell-derived neurotrophic factor (GDNF) and fibroblast growth factors (FGFs), which maintain a stem-cell phenotype (Ishii et al., 2012; Sharma and Braun, 2018; Kitadate et al., 2019). GDNF is produced by several somatic cell types, including Sertoli, peritubular myoid and testicular endothelial cells (Meng et al., 2000; Chen et al., 2014; Bhang et al., 2018), and it is secreted in a cyclic manner during adult spermatogenesis along the length of the seminiferous epithelium (Sharma and Braun, 2018). In mice, targeted transgenic overexpression of GDNF in spermatogonia is associated with accumulation of A_undiff_ and formation of cell clusters resembling seminomatous tumours (Meng et al., 2001). Further, GDNF overexpression in Sertoli cells results in formation of clusters of primitive GFRA1+ LIN28A- cells (Sharma and Braun, 2018). These models highlight the importance of limiting GDNF production to appropriately control SSC fate.

Several members of the TGFβ superfamily are implicated as key regulators of SSC and male germline differentiation including activin A and bone morphogenetic protein (BMPs) (Itman et al., 2006; Young et al., 2015). The widely expressed growth factor activin A (encoded by *Inhba*) is formed by the homodimerization of two Inhba subunits. It has important regulatory roles in fetal, postnatal and adult testes, and its production and actions are tightly controlled (Barakat et al., 2008; Itman et al., 2011; Mendis et al., 2011; Young et al., 2015; Wijayarathna and de Kretser, 2016). During fetal life, activin A governs development and proliferation of both germ and somatic cells, promoting proliferation of Sertoli cells and restricting germ cell proliferation; the outcome of dysregulated activin A bioactivity is an altered Sertoli:germ cell ratio and fetal steroidogenesis (Mendis et al., 2011; Whiley et al., 2020). Further consequences of low activin signalling (*Inhba* ^*BK*/BK^ mice) is the delayed onset of fertility, reinforcing the concept that activin A levels modulate the pace of testis development (Brown et al., 2000). *In vitro* studies have shown activin A supports differentiation of mouse (Nagano et al., 2003) and human SSCs (Tan et al., 2020a), however the effects of chronically elevated activin A on SSCs *in vivo* are unknown.

Inhibin ⍺ (encoded by *Inha*) forms Inhibin A when linked to an Inhba subunit and this is a potent inhibitor of activin A. As a consequence, mice lacking the inhibin ⍺ subunit (*Inha* KO) exhibit increased circulating activin A and B levels (>10 fold) (Matzuk et al., 1994). The *Inha* KO mouse model revealed a role for inhibin as a tumour inhibitor (Matzuk et al., 1992). Inhibin-deficient male mice with elevated activin A levels exhibit testicular enlargement, fewer Leydig cells and develop focally invasive, undifferentiated gonadal stromal tumours by weeks 4–5. In parallel with an increase in tumour mass, spermatogenesis deteriorates (Matzuk et al., 1992). Based on histological analyses, the tumours are considered to originate from Sertoli cells (Matzuk et al., 1992; Kumar et al., 1996), however the identity and features of these tumours has not been examined in detail. *Inha*/*Smad*3 double KO mice have reduced tumour frequency and size, confirming that elevated activin signalling via SMAD3 drives tumour formation (Li et al., 2007). This fits with their cell of origin being immature Sertoli cells, as SMAD3, but not SMAD2, is activated by activin A in these cells (Itman et al., 2009). Follicle Stimulating Hormone (FSH) specifically augments tumour progression during the second postnatal week of by promoting Sertoli cell proliferation, the sole testicular cell type bearing FSH receptors (Matzuk et al., 1994; Kumar et al., 1996; Haverfield et al., 2017). In general, tumour formation is associated with progressive acquisition of hallmark traits, including sustained cell proliferation, induced angiogenesis, increased immune cell activation and re-programming of energy metabolism; these collectively create a microenvironment that sustains tumour growth (Hanahan and Weinberg, 2011). In humans, testicular tumours derived from somatic cells are rare, representing ∼5% of all tumours in adults, but comprising ∼40% of cases in children (Rajpert-De Meyts et al., 2018). These cancers are generally benign and highly treatable, therefore the molecular and cellular landscape underpinning somatic cell tumours is not well studied.

In this study, we characterised the spermatogonial populations in mice with chronically elevated activin A levels (*Inha* KO) and identified an expanded population of SSCs and other A_undiff_. By examining the molecular landscape of the *Inha* KO somatic cell tumours and their surrounding tubules, we provide new insights into their cellular composition, establish a new understanding of the tumour microenvironment and show it can support SSCs. In discovering that these tumours, formed in mice with chronically elevated activin A, produce high levels of transcripts encoding growth factors that support SSC maintenance, we demonstrate the value of examining *Inha* KO mice to identify conditions that underpin SSC biology.

## Materials and methods

### Animals and genotyping

Mice were housed at the Monash Medical Centre Animal Facility with a 12h light/dark cycle and food and water available *ad libitum*. All animal procedures were carried out in accordance with the Australian Code of Practice for the Care and Use of Animals for Scientific Purposes. This study was approved by the Monash University Animal Ethics Committee. Mice lacking the inhibin alpha subunit (Matzuk et al., 1992), termed *Inha* KO (C57/Bl6 background), were maintained through heterozygote breeding. Adult *Inha* KO male mice were collected at postnatal (P) day 48–64. Tails were collected from each animal for commercial genotyping (Transnetyx).

### Histological analyses

Whole mouse testes were weighed, and fixed in 4% paraformaldehyde (PFA, ThermoFisher Scientific) overnight at 4°C. Testes were washed 3 × 15 min in PBS, cut in half and cryoprotected in 30% (w/v) sucrose for 2–3 days at 4°C. Testis tissues were then embedded in Tissue-Tek O.C.T. Compound (Sakura) and stored at −80°C until sectioned. Frozen sections (7 µm) were cut onto Superfrost Plus slides by Monash Histology Platform staff (MHTP node). For immunofluorescent analyses, frozen sections were air dried for 15–20 min then washed in PBS, 3 × 10 min. Sections were circled with a wax pen (Cedarlane Laboratories), then blocked with 10% (v/v) fetal calf serum (FCS, Bovogen) in 2% (w/v) bovine serum albumin (BSA, Sigma-Aldrich) in PBS for >1 h. Primary antibodies diluted in blocking solution were used to detect the proteins listed in Table 1. Slides were placed in a humid chamber overnight at 4°C. The next day, slides were washed in PBS 3 × 8 min, and secondary antibodies (Invitrogen, donkey anti-goat 488, A11055, RRID:AB_2534102; donkey anti-rabbit 555, A31572, RRID:AB_162543; Jackson ImmunoResearch Labs, donkey anti-rat 647, 712–605–153, RRID:AB_2340694) diluted 1:500 in 2% BSA/PBS were applied to sections and incubated for 2 h at room temperature in a humid chamber. Slides were washed in PBS 3 × 8 min, then sections were counterstained with 5 μg/mL DAPI (Sigma-Aldrich) prior to mounting with Prolong Gold mounting medium (Invitrogen) under glass coverslips. Slides were scanned (Monash Histology Platform, MHTP node) using the Olympus VS.120^®^ Virtual Slide Microscope System. For Hematoxylin and Eosin (H&E) staining, adult mouse testes were fixed in Bouins solution for 4–5 h, washed in 70% ethanol (v/v) 2 × 5 min, then processed, embedded in paraffin, and sectioned (4 µm) prior to H&E staining (Monash Histology Platform, MHTP node). H&E sections were imaged using an Olympus BX53 microscope with Olympus DP73 camera and Olympus cellSens Software (RRID:SCR_014551).

**TABLE 1:** Antibody details.

Antibody	Species	CAT #	Supplier	Dilution	RRID
SALL4	Rabbit	ab29112	Abcam	1:250	RRID:AB_777810
GFRA1	Goat	AF560	R&D Systems	1:250	RRID:AB_2110307
KI67	Rat	14–5698–82	Invitrogen	1:250	RRID:AB_10854564
EOMES	Rabbit	MAB8889	R&D Systems	1:250	NA
GILZ	Rat	14–4033–82	Invitrogen	1:250	RRID:AB_1311225
LIN28A	Rabbit	#8641	Cell Signalling	1:500	RRID:AB_10997528
GCNA	Rat	ab82527	Abcam	1:500	RRID:AB_1659152
SOX3	Goat	AF2569	R&D Systems	1:250	RRID:AB_2239933
LAMININ	Rabbit	L9393	Sigma-Aldrich	1:200	RRID:AB_477163
CYP11A1	Rabbit		gift from A/Prof. Dagmar Wilhelm Svingen et al. (2012)	1:250	NA
PECAM	Rat	553370	BD Pharmingen	1:200	RRID:AB_394816
CD45	Rat	05–1416	Merck Millipore	1:800	RRID:AB_10562966

NA: RRID not available.

### Counting strategy

Centrally located testicular cross sections from 3 independent animals/genotype were assessed for each measured parameter. Three groups of adult seminiferous tubules were defined based on the mouse genotype and the proximity of the tubules to tumours. Tubules from *Inha* WT mice exhibited complete, normal spermatogenesis, as expected and were designated WT. In *Inha* KO testes, tubules distant from tumour foci exhibiting grossly ‘normal’ spermatogenesis were designated N, and tubules adjacent to tumours exhibiting spermatogenic deterioration were designated TAT (for tumour associated tubules). The perimeter of intact tubules (identified as those with an undamaged basement membrane) were measured (µm) using Fiji (Version 2.1.0, RRID:SCR_002285) (Schindelin et al., 2012) or QuPath (RRID:SCR_018257) (Bankhead et al., 2017) and counted. The tumour regions lacked intact tubules and were excluded from the quantitative IF analyses. Quantitative analyses of cells expressing proteins of interest was performed using panels of antibodies as follows: A) To assess the number of SSCs and their proliferation, all spermatogonia were identified using SALL4, then SALL4+ cells with GFRA1 and/or KI67 signals were counted. B) To assess the number of progenitor cells, all spermatogonia positive for both GILZ and SOX3 were counted. C) SOX9+ Sertoli cell nuclei were detected and counted using the Positive cell detection command in QuPath. A scaling range for each antibody was established based on settings that yielded no visible signal in the negative controls lacking primary antibody (secondary antibody only). Sertoli cell-only cords with no visible lumen and rete testis were excluded from all analyses.

### Flow cytometry

To generate single-cell suspensions from adult *Inha* WT and KO testes (without tumours, *Inha* KO ‘normal’ tubules only), dissected tissues were coarsely minced and washed with PBS, then digested with 1 mg/mL collagenase Type IV (Sigma-Aldrich) in DMEM medium (Gibco) and 50 μg/mL DNase I (Sigma-Aldrich). Tubule fragments were washed in PBS to deplete interstitial and peritubular myoid cells then dissociated with 0.25% trypsin/DMEM in the presence of DNase I and passed through a 70 μm cell strainer. For intracellular staining, cell suspensions were fixed in 4% PFA for 10 min at 37°C then permeabilized in methanol overnight at −20°C. Fixed and permeabilized cells were washed in PBS with 2% FCS then stained with the following antibodies diluted in PBS with 2% FCS: eFluor 450-conjugated anti-KI67 clone SolA15 (Thermo Fisher Scientific, 1:500, 48–5698–82, RRID:AB_11149124) and phycoerythrin (PE)-conjugated anti-KIT clone 2B8 (Thermo Fisher Scientific, 1:250, 12–1171–81, RRID:AB_465812), rabbit anti-mouse EOMES clone 1219A (R&D Systems, 1:1,000) and Alexa 647-conjugated anti-PLZF clone 9E12 (Hobbs et al., 2010). EOMES antibody was detected with Alexa 488-conjugated secondary antibody (Thermo Fisher Scientific and Jackson ImmunoResearch, 1:500). Cells were analysed on an LSR Fortessa (Monash Flowcore) and data processed with FlowJo software.

### Microdissection

Tissues were collected from decapsulated testes from *Inha* WT and KO mice aged between P53-55. Three discrete regions were microdissected from *Inha* KO testes: *Inha* KO ‘normal’ tubules (N), ‘tumour associated tubules’ (TAT), and tumour (TM) regions. *Inha* KO ‘normal’ seminiferous tubules were collected from areas distant from tumour regions. *Inha* KO tumour associated tubules ‘TAT’ and tumour regions ‘TM’, readily identified by the high level of vascularisation, were microdissected and snap frozen. To assess heterogeneity of multiple tumours within a single testis and between testes from the same animal, ‘normal’, ‘TAT’ and multiple ‘tumour’ regions from both testes of a single mouse were analysed.

### RNA sequencing

Testis tissues (18–100 mg) were homogenised using a syringe and 23G needle. RNA was isolated from *Inha* WT mouse testis tubules (*n* = 3), and *Inha* KO ‘normal’ (*n* = 4), ‘tumour associated tubules’ (*n* = 4) and tumour regions (*n* = 6) using the RNeasy Mini Kit (Qiagen) with on-column DNase treatment (RNase-free DNase Set, Qiagen) according to the manufacturer’s instructions. RNA integrity and concentration were assessed using the 2100 Bioanalyser (Agilent) prior to RNA sequencing (RIN values: 8.3–9.3). RNA-Seq was performed using a custom in-house multiplex method, modified from (Grubman et al., 2021) (MHTP Medical Genomics Facility). Briefly, samples were given a unique i7 index (together with UMI) during individual pA priming and first strand synthesis which also adds a template switch sequence to the 5′-end. Samples were then pooled and amplified using P7 and an oligo which binds the template switch sequence. Final library construction was completed by tagmentation and addition of P5 (with i5 index) by PCR. Sequencing was performed on an Illumina NSQ2k run with up to 101 nt SR (cDNA). An 18 nt i7 read contains the 8 nt index and 10 nt UMI and, where required, an 8 nt i5 index read is also generated.

Bioinformatics analysis was performed as follows: Raw fastq files were analysed using the nf-core/rnaseq pipeline (Ewels et al., 2020) using STAR aligner (RRID:SCR_004463) (Dobin et al., 2013) to map the raw reads to GRCm38 (*Mus musculus*) reference genome. Reads were quantified using Salmon (RRID:SCR_017036) (Patro et al., 2017) producing the raw genes count matrix. Alignment and transcript quantification were completed via the Laxy platform (Perry and Powell, 2020). Raw counts were exported to and analysed with Degust (RRID:SCR_001878) (Powell et al., 2019), a web tool which performs differential expression analysis using limma voom normalisation (Law et al., 2014), producing counts per million (CPM) library size normalisation and trimmed mean of M values (TMM) normalisation (Robinson and Oshlack, 2010) for RNA composition normalisation. This identified differentially expressed genes (DEGs) in WT and *Inha* KO ‘normal’ (N), tumour associated tubules (TAT) and tumour (TM) regions using the following criteria: false discovery rate (FDR) < 0.05, Abs log fold change (FC) > 1.5X. RNA-seq data are available via GSE236488.

To identify enriched biological themes, we performed DAVID analysis (RRID:SCR_001881) (Huang da et al., 2009a; Huang da et al., 2009b) which requires a list of <3,000 DEGs. To accommodate this requirement, FDR and Abs log FC values were adjusted for each group as follows: WT and KO N; FDR cut-off <0.05, Abs log FC > 1.5X, WT and KO TAT; FDR cut-off <0.01, Abs log FC > 1.5X, WT and KO TM; FDR cut-off <0.0001, Abs log FC > 3X. Ingenuity Pathway Analysis software (IPA, RRID:SCR_008653) (Qiagen, Hilden, Germany) was used to identify downstream Disease and Bio Functions in adult *Inha* KO N, TAT and TM samples compared to WT (Kramer et al., 2014). RNAseq data from each comparison was uploaded to the Ingenuity Knowledge Base (Genes Only) reference set, using Expr Fold Change range: < −3; > +3, and Expr *p*-value of 0.01. Significance calculated using the right-tailed Fisher’s exact test.

## Results

### Increased number and proliferation of SSCs and spermatogonia in adult *Inha* KO testes

**FIGURE 1:**
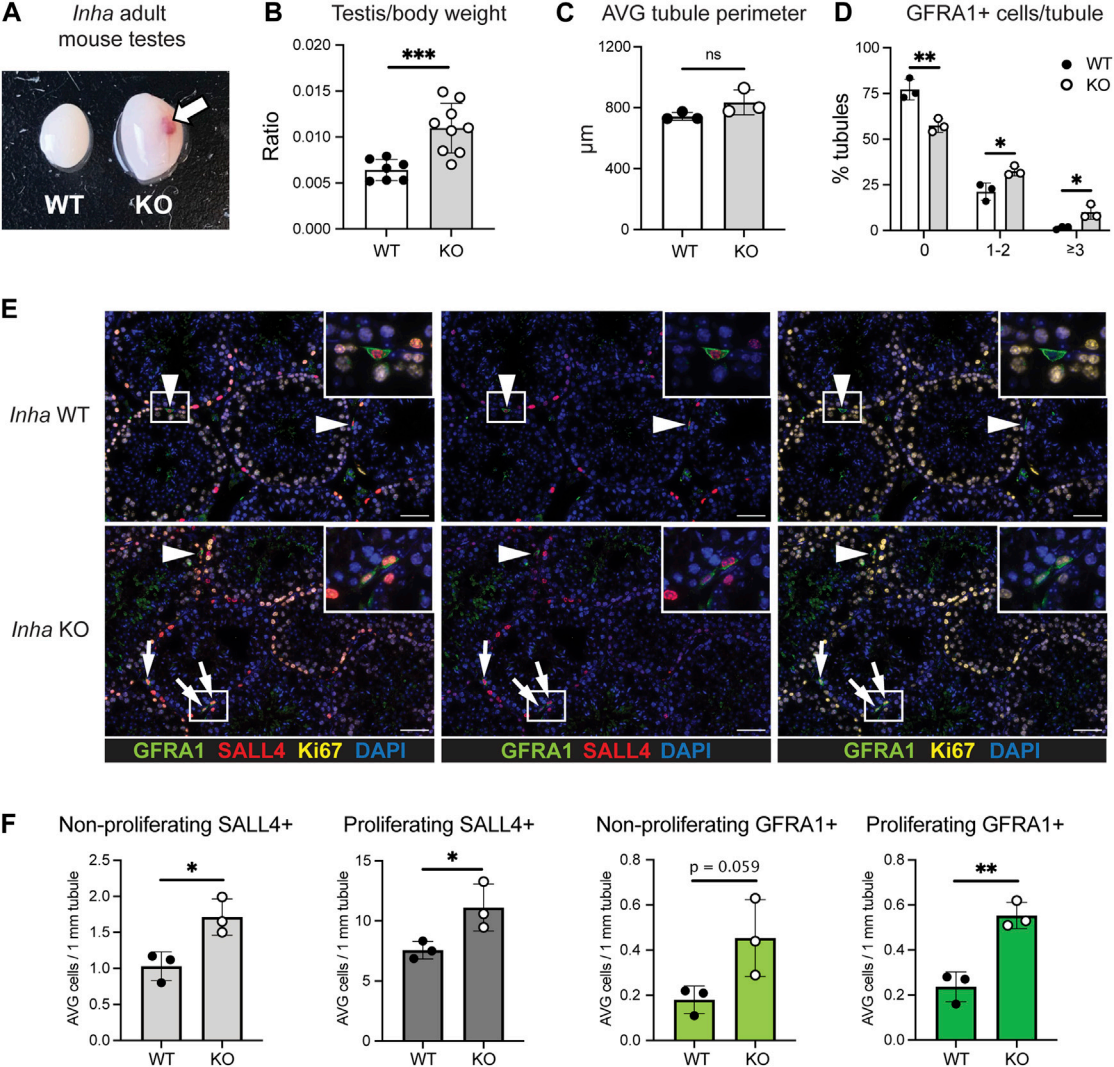
Increased number of proliferative SALL4+ and GFRA1+ spermatogonia in ‘normal’ tubules from adult *Inha* KO mouse testes. **(A)** Gross comparison of adult *Inha* WT and KO mouse testes. Haemorrhagic region indicated by arrow. **(B)** Testis/body weight ratio, **(C)** Average cord perimeter, and **(D)** Percentage of tubules with 0, 1-2, or ≥3 GFRA1+ cells/tubule in adult *Inha* WT and KO ‘normal’ testes. **(E)** Representative images of adult *Inha* WT and KO testes. White arrow heads indicate non-proliferating GFRA1+ spermatogonia; white arrows, proliferating GFRA1+ spermatogonia, scale bar = 50 µm. **(F)** Quantification of SALL4+ spermatogonia and GFRA1+ cell populations and their proliferation (KI67) status in adult (P48-50) *Inha* WT and KO testes. Tubule perimeter measurements and cell counting performed on *Inha* WT and KO adult animals (*n* = 194–304 tubule cross-sections from n = 3 animals/genotype). All graphs show mean ± SD. Significance determined using an unpaired two-tailed t-test, *p < 0.05, **p < 0.01, ***p < 0.001.

In mice, genetic deletion of the *Inha* gene (*Inha* KO) results in testicular enlargement (Figures 1A,B), and development of focally invasive tumours by approx. 4 weeks of age (Figure 1A, arrow) (Matzuk et al., 1992; Matzuk et al., 1994; Coerver et al., 1996). Although reduced activin A levels during fetal life affect testis cord development (Archambeault and Yao, 2010; Moody et al., 2022), in adulthood elevated activin A bioactivity does not significantly affect average tubule perimeter in grossly ‘normal’ tubule cross-sections from *Inha* KO mice (Figure 1C). Given that spermatogonia reside on the basement membrane, all cell counts were normalised to tubule perimeter.

To understand the impact of chronically elevated activin A on spermatogonial populations, we first evaluated the incidence of SALL4+ (marks all spermatogonia, SPG), GFRA1+ (SSC-enriched population) and KI67+ (proliferation) cells in adult *Inha* KO ‘normal’ tubules. The percentage of tubule cross-sections containing no GFRA1+/SALL4+ cells was significantly lower, and those containing 1-2 or ≥3 GFRA1+/SALL4+ cells was significantly higher, compared to wildtype (WT) testes (Figure 1D). The overall abundance of SALL4+ SPG and GFRA1+/SALL4+ SPGs in *Inha* KO testes was significantly increased (by 50% and 140%, respectively), and each population exhibited a higher incidence of KI67+ cells, indicating enhanced proliferation (Figures 1E, F). Additionally, in WT testes, there was approximately 1 GFRA1+ cell per 21 SPGs, however in the KO testes, there was approximately 1 GFRA1+ cell per 13 SPGs, suggesting a higher proportion of SPG were SSCs in ‘normal’ tubule cross-sections of adult *Inha* KO mice compared to in WT controls.

**FIGURE 2:**
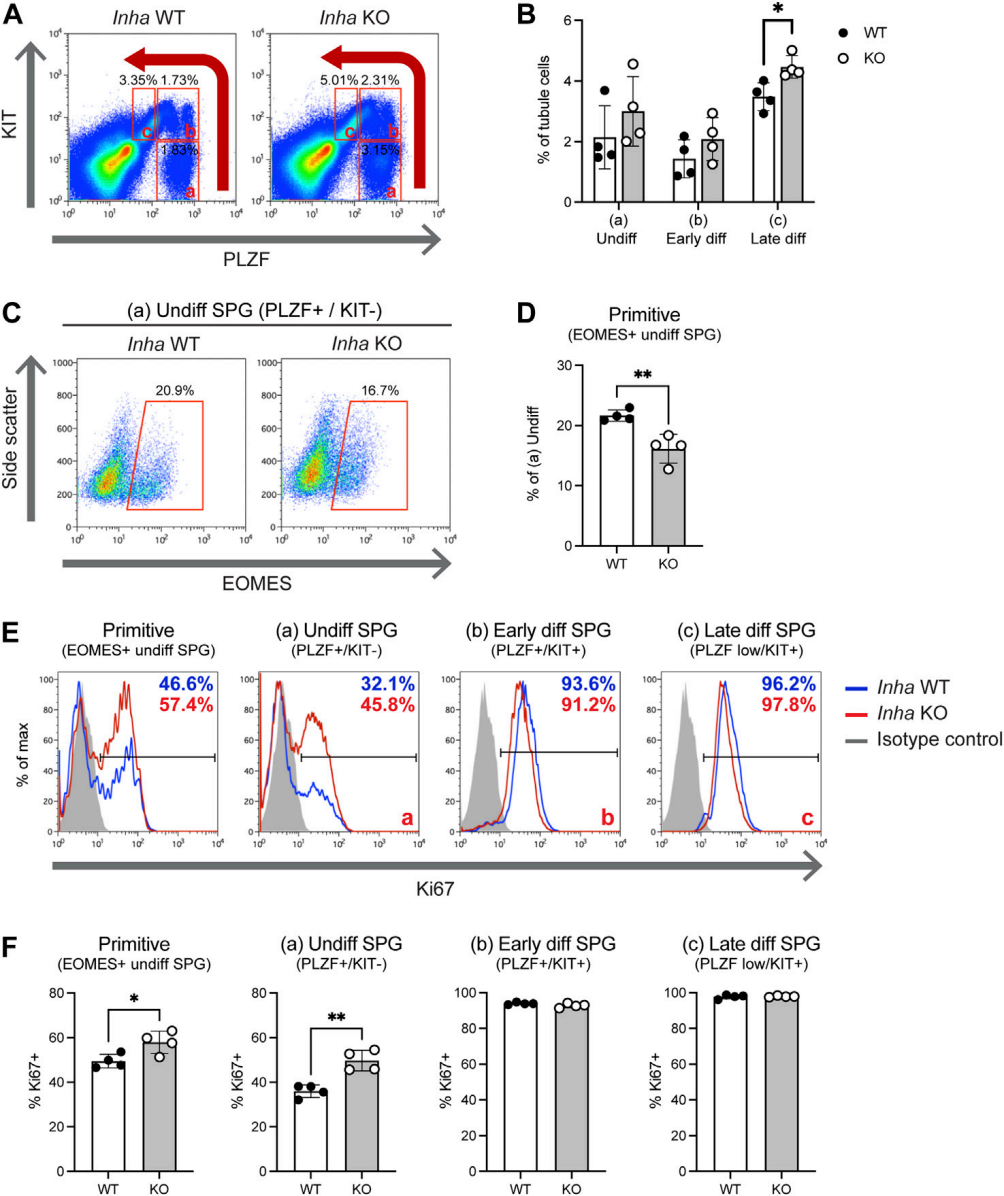
Flow cytometry analysis of testis tubule cells from adult *Inha* WT and KO ‘normal’ testes. **(A)** Gating strategy used to identify (a) undifferentiated SPG (PLZF+/KIT–), (b) early differentiating SPG (PLZF+/KIT+), and (c) late differentiating SPG (PLZF^low^/KIT+) cells. Percentages of cells within gates are indicated. **(B)** Graph showing subpopulations (a–c) of cells as a percentage of tubule cells (interstitial depleted). **(C)** Gating strategy for EOMES+ cells within the PLZF+/KIT- population. **(D)** Graph showing EOMES+ fraction as a percentage of (a) undifferentiated cells. **(E)** Representative flow cytometry and **(F)** graphs showing proportion of proliferating (KI67+) cells in populations from **(A)** and **(C)**. All graphs show mean percentage of cells in each population ± SD (*n* = 4 animals/genotype). Significance determined using a two-tailed unpaired t-test, *p < 0.05, **p < 0.01.

Next, we characterised populations of SPG isolated from adult WT and *Inha* KO ‘normal’ testes (without visible tumours) by intracellular staining for PLZF, KIT and EOMES by flow cytometry (Chan et al., 2017; La et al., 2018b). Undifferentiated SPG are PLZF+/KIT- (population a), early differentiating (diff) SPG are PLZF+/KIT+ (b), and late differentiating SPG are PLZF^low^/KIT+ (c) (Figures 2A, B). The relative abundance of undiff SPG (a) and early diff SPG (b) was variable and not significantly altered, however late diff SPG (c) were significantly increased (Figure 2B), consistent with normal differentiation from an elevated population of undiff SPG. Primitive SPG are EOMES+ and were identified in the (a) undiff SPG population (Figure 2C) and intriguingly, the proportion of undiff cells that were EOMES+ was significantly decreased in *Inha* KO testes (Figure 2D). In agreement with our previous data, primitive and undiff SPG were more proliferative, as indicated by KI67 (Figures 2E, F). These data indicate long term exposure to elevated activin A levels leads to altered SSC and spermatogonial populations.

### Clusters of GFRA1+ spermatogonia in tubules adjacent to tumour regions

**FIGURE 3:**
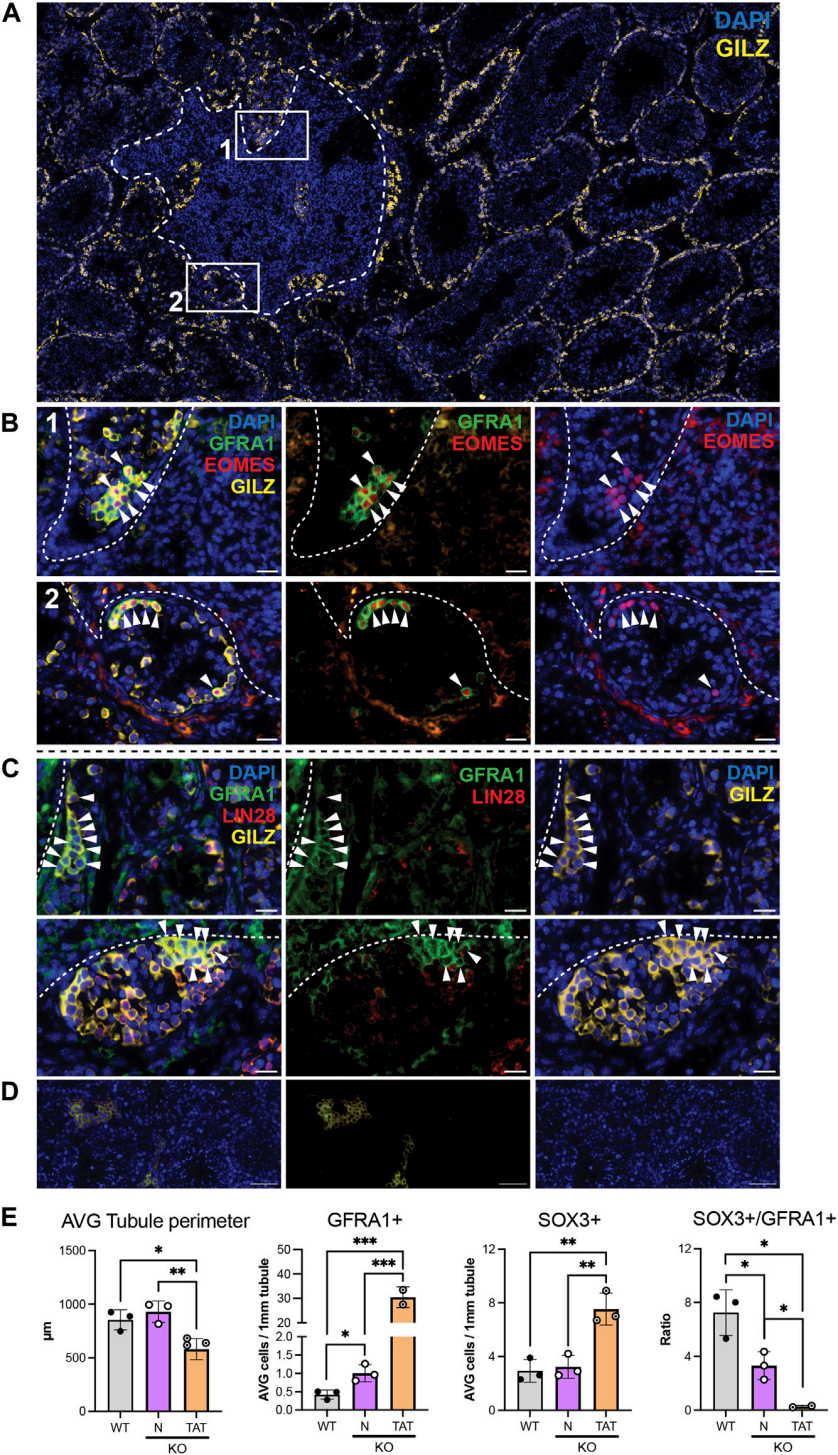
Tubules adjacent to stromal tumour regions are associated with clusters of cells with SSC characteristics. **(A)** Representative image of tumour region (white dashed line) in adult *Inha* KO testes. Numbered boxes show location of tumour associated tubules (designated TAT) (in B) relative to tumorigenic region. **(B)** Representative images showing clusters of GFRA1+/EOMES+ cells. White arrow heads indicate GFRA1+/EOMES+/GILZ+ spermatogonia. **(C)** High density GFRA1+ cells are LIN28– or low. White arrow heads indicate GFRA1+/LIN28-/GILZ+ spermatogonia. **(D)** Representative negative control images. Scale bars: main image = 200 μm, panels **(B–C)** = 20 μm, negative control panels = 50 µm. **(E)** Graphs show average (AVG) tubule perimeter (µm), AVG number of GFRA1+ cells and SOX3+ cells/mm tubule perimeter, and ratio of SOX3+/GFRA1+ cells in *Inha* WT (grey bars) and KO ‘normal’ (N, purple bars) and TAT tubules (orange bars). All graphs show mean ± SD (n = 3/genotype). Significance determined using a two-tailed unpaired t-test, *p < 0.05, **p < 0.01, ***p < 0.001.

In WT testis cross-sections, GFRA1+ cells are relatively rare and often observed as isolated SPG on the basement membrane. Therefore, the finding of GFRA1+ cell clusters in seminiferous tubules adjacent to tumour regions (termed here ‘TAT’, for tumour associated tubules) was intriguing (Figures 3A–C). Theses clusters were frequently displaced from the basement membrane, and further examination revealed a high proportion of these cells were EOMES+ (Figure 3B, panels 1–2) and LIN28A– or LIN28A low (Figure 3C). EOMES expression identifies a discrete population of A_s_-A_pr_ spermatogonia (Sharma et al., 2019), and LIN28A is reported to be absent or weakly expressed in A_s_ and may be expressed at either low or high levels in A_pr_ spermatogonia (La et al., 2018b; Sharma and Braun, 2018; McAninch et al., 2020). Thus, this combination of staining (GFRA1+/EOMES+/LIN28A- or LIN28A low) indicates these clusters are comprised of A_s_ – A_pr_ spermatogonia. To establish if SSC clusters result from a failure to differentiate, we applied the progenitor marker SOX3 and the differentiation marker KIT. Both SOX3+ and KIT+ cells were present in TAT tubules (Supplementary Figure S1A, B) demonstrating SSC differentiation was occurring. Next, we reasoned that the relative proportion of SOX3+ cells compared to GFRA1+ SPGs would be significantly reduced if SSC differentiation was impeded, so we quantified SOX3+/GILZ+ cells in WT, *Inha* KO N and TAT tubules (Figure 3E). In *Inha* KO ‘normal’ N tubules (Figure 3E, purple bars) compared to WT, the average tubule perimeter was not different, the average number of GFRA1+ cells/1 mm tubule perimeter was significantly increased in *Inha* KO N tubules, however SOX3+ cell numbers were unchanged. In *Inha* KO TAT tubules (Figure 3E, orange bars), the average tubule perimeter was reduced compared to WT and KO N, consistent with tubules exhibiting severely disrupted spermatogenesis. The average number of GFRA1+ cells/1 mm tubule perimeter was further increased in TAT tubules, compared to WT and KO N, while SOX3+ cells were also significantly increased. The ratio of SOX3+/GFRA1+ cell was significantly lower in *Inha* KO N and TAT tubules compared to WT, suggesting SSC differentiation is suppressed in *Inha* KO testes (Figure 3E). Thus, the presence of clusters of A_undiff_ resembling primitive SSCs along with a proportional decrease in progenitors indicates SSC differentiation is hindered in TAT tubules.

### Transcriptional dysregulation of seminiferous tubules increases in parallel with tumour proximity

**FIGURE 4:**
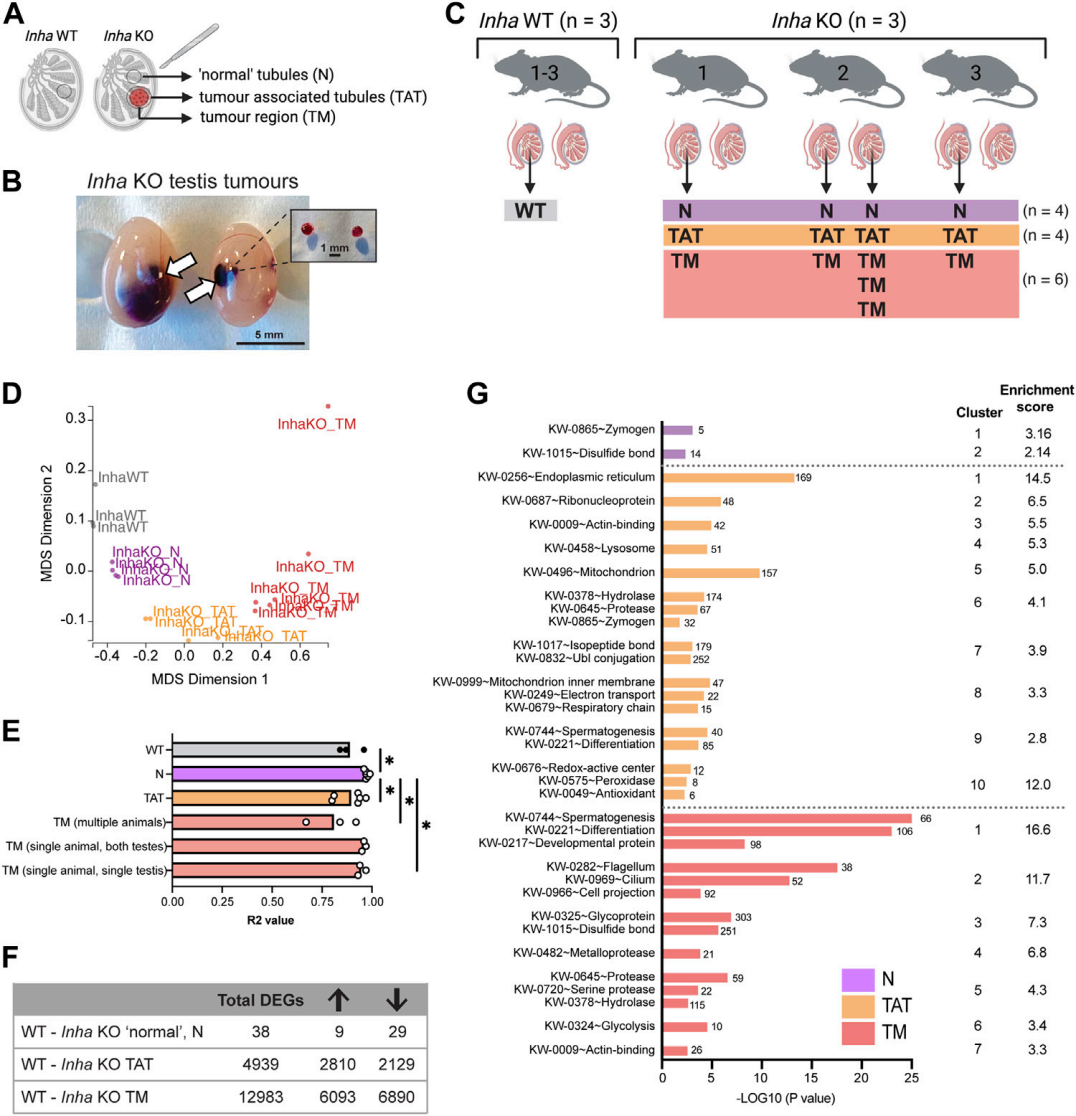
Transcriptional analyses of *Inha* WT and microdissected *Inha* KO ‘normal’ (N), tumour associated tubules (TAT) and tumour (TM) regions. **(A)** Diagram illustrating *Inha* WT and KO ‘normal’ N, TAT and TM regions. **(B)** Single and multiple tumours in *Inha* KO adult testes, *in situ* and after dissection. **(C)** Experimental overview of sample collections and microdissected regions; *Inha* WT tubules (grey) and *Inha* KO ‘normal’ N (purple), *Inha* KO ‘tumour associated tubules’ TAT (orange), *Inha* KO ‘tumours’ TM (red) (N = 3-6 samples/group). **(D)** MDS Dimension plot demonstrating groups are transcriptionally distinct from each other. **(E)** Coefficient of determination (R^2^ values) indicates the degree of similarity between biological replicates in each group and shows *Inha* KO TMs from multiple animals are most transcriptionally heterogeneous. Calculations performed on CPM for the entire RNAseq dataset (∼22,000 genes). **(F)** Table showing the total number of differentially expressed genes (DEGs) for each KO region relative to the WT control (FDR <0.05, Abs logFC >1.5X). Arrows indicate number of DEGs increased and decreased. **(G)** Graph shows top biological processes (UniProt keyword) affected in *Inha* KO ‘normal’, TAT and TM regions identified using DAVID analysis.

Previous histological analyses described *Inha* KO tumours as undifferentiated gonadal stromal tumours derived from cells of the Sertoli cell lineage (Matzuk et al., 1992; Matzuk et al., 1994) that are positive for GATA4 (Sertoli cell nuclei) (Haverfield et al., 2017). To explore the molecular features of these tumours in *Inha* KO testes, we performed RNAseq on *Inha* WT tubules, microdissected *Inha* KO ‘normal’ (N), tumour associated tubules (TAT) and tumour regions (TM) (Figure 4A). The presence of multiple tumours within a single testis (Figure 4B) allowed tumour heterogeneity to be compared across 3 independent animals, as well as within a single animal and a single testis (Figure 4C). A multidimensional scaling (MDS) plot (Figure 4D) identified that all 4 groups were transcriptionally distinct from each other, indicating the microdissection was effective at separating distinct regions. To examine the degree of tumour heterogeneity, the coefficient of determination (R^2^ value) of the global transcriptome (CPM of ∼22,000 genes/sample) was calculated between samples from each group (WT and *Inha* KO ‘N’, ‘TAT’ and ‘TM’, including single tumours from multiple animals (multiple animals), tumours from both testes from the same animal (single animal, both testes), and tumours within a single testis (single animal, single testis) (Figure 4E). The overall heterogeneity of WT testis samples was low, with R^2^ values between 0.84–0.96. In *Inha* KO testes, heterogeneity was even lower in ‘normal’ tubules and in tumours found within a single animal (R^2^ values: 0.93–0.99), suggesting a high degree of transcriptional similarity between focal tumours within testes from an individual mouse. The greatest heterogeneity was observed between tumours from multiple animals (R^2^ values: 0.67–0.92). During the course of cancer development, cellular heterogeneity generally increases (Marusyk and Polyak, 2010), therefore this heterogeneity may reflect the varying severity of the tumours analysed.

Overall, we found that compared with *Inha* WT controls, the number of differentially expressed genes (DEGs) in each KO group increased in parallel with tumour proximity (Figure 4F). As expected, *Inha* was significantly decreased in all *Inha* KO groups and *Inhba* (encoding activin A) was increased in both the TAT and TM groups (Supplementary Figure S2). In *Inha* KO ‘normal’ testis tubules, there were 38 DEGs compared to *Inha* WT testes (FDR <0.05, absolute fold change ≥1.5, Supplementary Table S1) including key Leydig cell-associated genes (*Insl3*, *Cyp17a1*, *Hsd17b3*, decreased 3 to 5-fold). Using publicly available testis single-cell RNAseq datasets (Hermann et al., 2018; Tan et al., 2020b), we found 37% (14/38) of the DEGs are normally exclusively expressed in Leydig cells and all were decreased, including several members of the kallikrein 1-related peptides (*Klk1b22*, *Klk1b21*, *Klk1b24*, *Klk1b27*, decreased ∼ 5-fold). The reduced expression of all Leydig cell-specific DEGs in the *Inha* KO testes most likely reflects their reduced number (Matzuk et al., 1992). DAVID analysis identified the top affected biological processes to be enriched for the terms ‘Zymogen’, referring to the enzymatically inactive precursor of mostly proteolytic enzymes, and ‘Disulphide bond’, both of which include the kallikrein 1-related peptide genes (Figure 4G, purple bars). To further understand features of the KO ‘normal’ regions, Ingenuity Pathway Analyses (IPA) was employed and identified cellular movement, immune cell trafficking, inflammatory response, lipid metabolism and cancer in the top 20 diseases and bio functions (Supplementary Table S2). Importantly, these indicate the pathways influenced by the subtle transcriptional changes occurring in the *Inha* KO N tubules, which precede their evolution into the more severe phenotypes evident in TAT and TM regions.

The TAT samples comprised seminiferous tubules and interstitial areas bordering tumour regions. In this group, 4,939 DEGs were identified compared to WT control testis (Supplementary Table S1). DAVID analysis revealed significant enrichment for terms including endoplasmic reticulum (ER), ribosomal proteins and mitochondrion (Figure 4G, orange bars). During tumorigenesis, the high proliferation rate of cancerous cells requires increased ER protein assembly, folding and transport, which can induce physiological ER stress (Chen and Cubillos-Ruiz, 2021), and increased mitochondrial metabolism (Lee et al., 2022). Our data showing 30 ribosomal and 5 mitoribosomal transcripts were significantly increased in TAT samples (Supplementary Table S1) provides evidence that organelles in cells near to these testicular tumours have adopted a stress-response phenotype. In agreement with this, IPA analyses, identified Metabolic disease in the top 20 Disease and Bio Function categories (Supplementary Table S2).

In the tumour regions (TM), we identified 12983 DEGs *versus* WT testis (Supplementary Table S1) associated with UniProt keywords such as spermatogenesis, cell differentiation, motile cilium and metalloprotease (Figure 4G, red bars, DAVID analysis). IPA analysis further identified the top Disease and Bio Functions as Cancer, Organismal Injury, and Abnormalites, and Endocrine System disorder (Supplementary Table S2). Reproductive related categories included Reproductive System Disease and Reproductive System Development and Function (Supplementary Table S2). This confirms the tumour regions assume a transcriptional landscape consistent with disease and loss of spermatogenesis.

### *Inha* KO tumours possess some Sertoli cell features and have a striking steroidogenic phenotype

**FIGURE 5:**
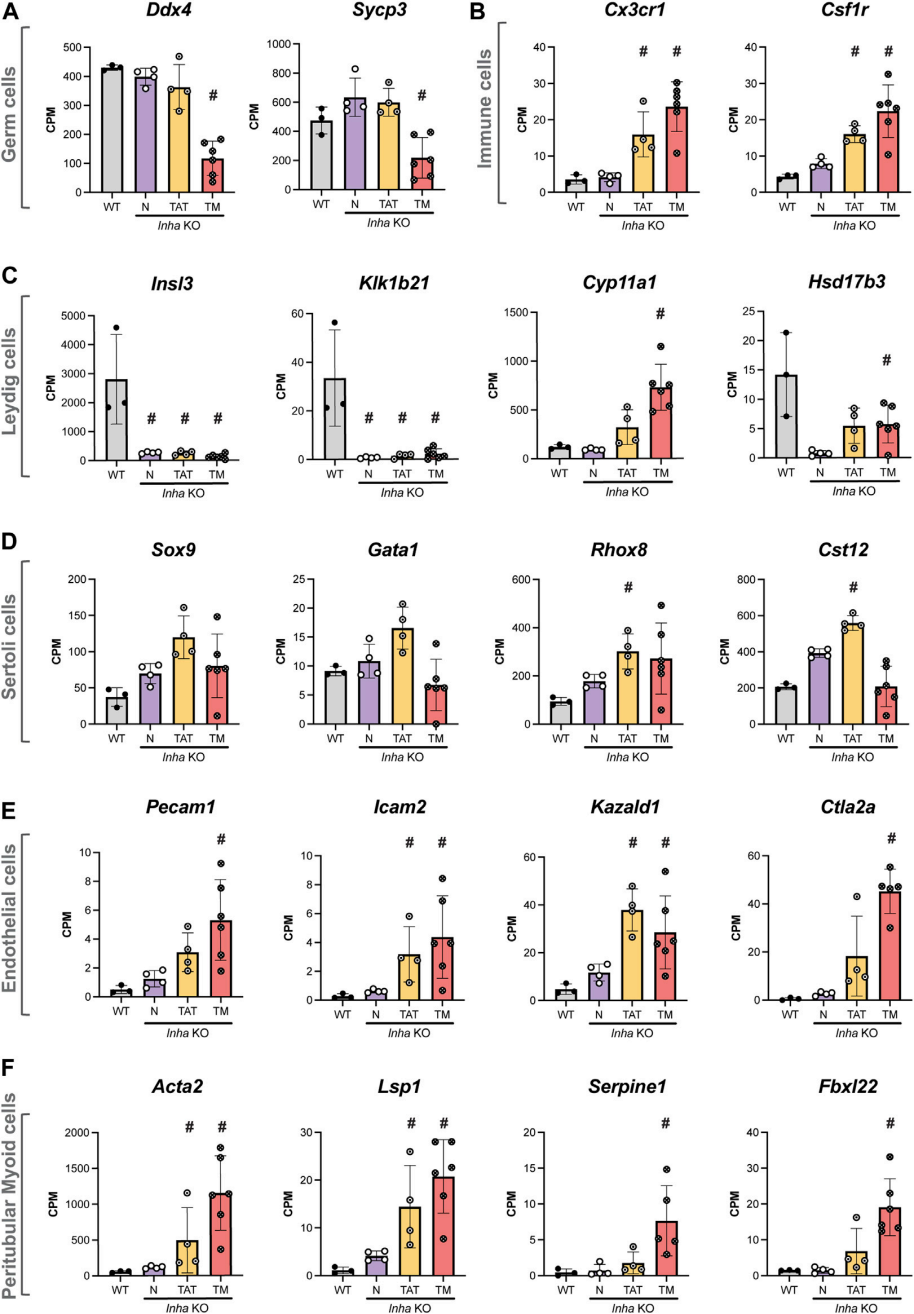
Transcriptional profile of major testicular cell types in adult *Inha* WT and KO tumours. Transcripts enriched in **(A)** germ cells, **(B)** immune cells, **(C)** Leydig cells, **(D)** Sertoli cells, **(E)** endothelial cells and **(F)** peritubular myoid cells. All graphs show Counts Per Million (CPM) from RNAseq analysis of *Inha* WT tubules (grey) and N, *Inha* KO ‘normal’ (purple), TAT, *Inha* KO ‘tumour associated tubules’ (orange), TM, *Inha* KO ‘tumours’ (red) (n = 3–6/group). Mean ± SD. Significance (indicated by #) compared to WT determined in DEGUST (FDR <0.05, Abs logFC >0.05).

**FIGURE 6:**
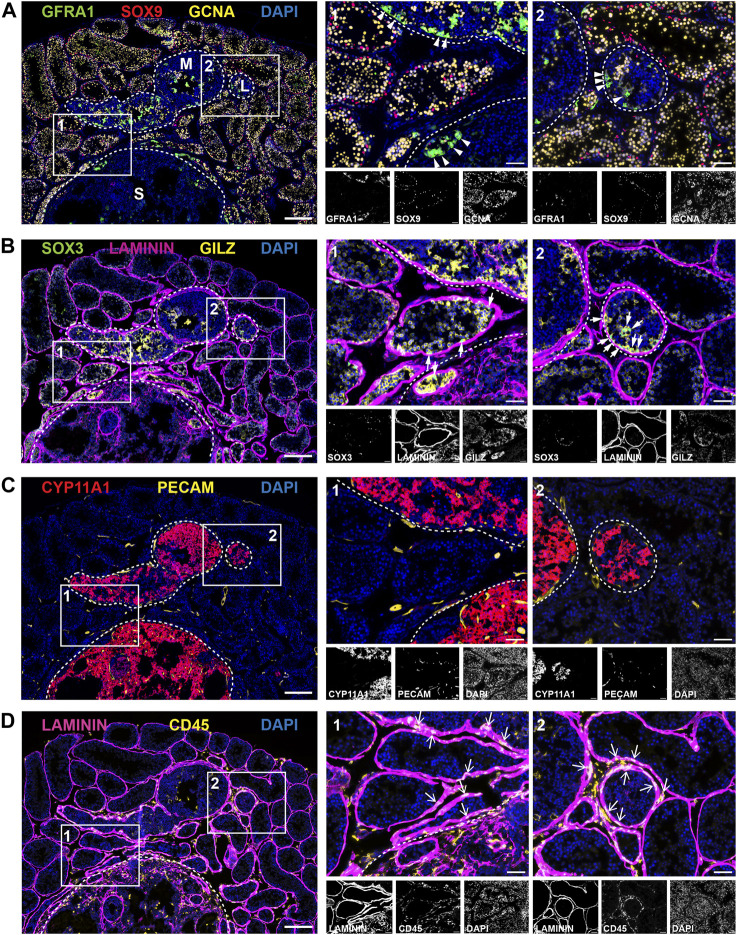
Cellular landscape of tumour regions in adult *Inha* KO testes. Panels **(A–D)** show representative images of different antibody combinations over the same tumour regions; **(A)** GFRA1 (SSC-enriched population), SOX9 (Sertoli cells), GCNA (all germ cells), **(B)** SOX3 (progenitor SPG), LAMININ (basement membrane), GILZ (SPG and early meiotic cells), **(C)** CYP11A1 (Leydig cells/steroidogenic), PECAM (endothelial cells/vasculature) and **(D)** LAMININ (basement membrane), CD45 (immune cells). Tumour regions designated low (L), medium (M) or severe (S) grade and are outlined with white dotted line. High magnification ROIs are shown with a solid white line and are numbered 1 or 2. Scale bars = 200 µm (low magnification image, left) or 50 µm (all other images).

**FIGURE 7:**
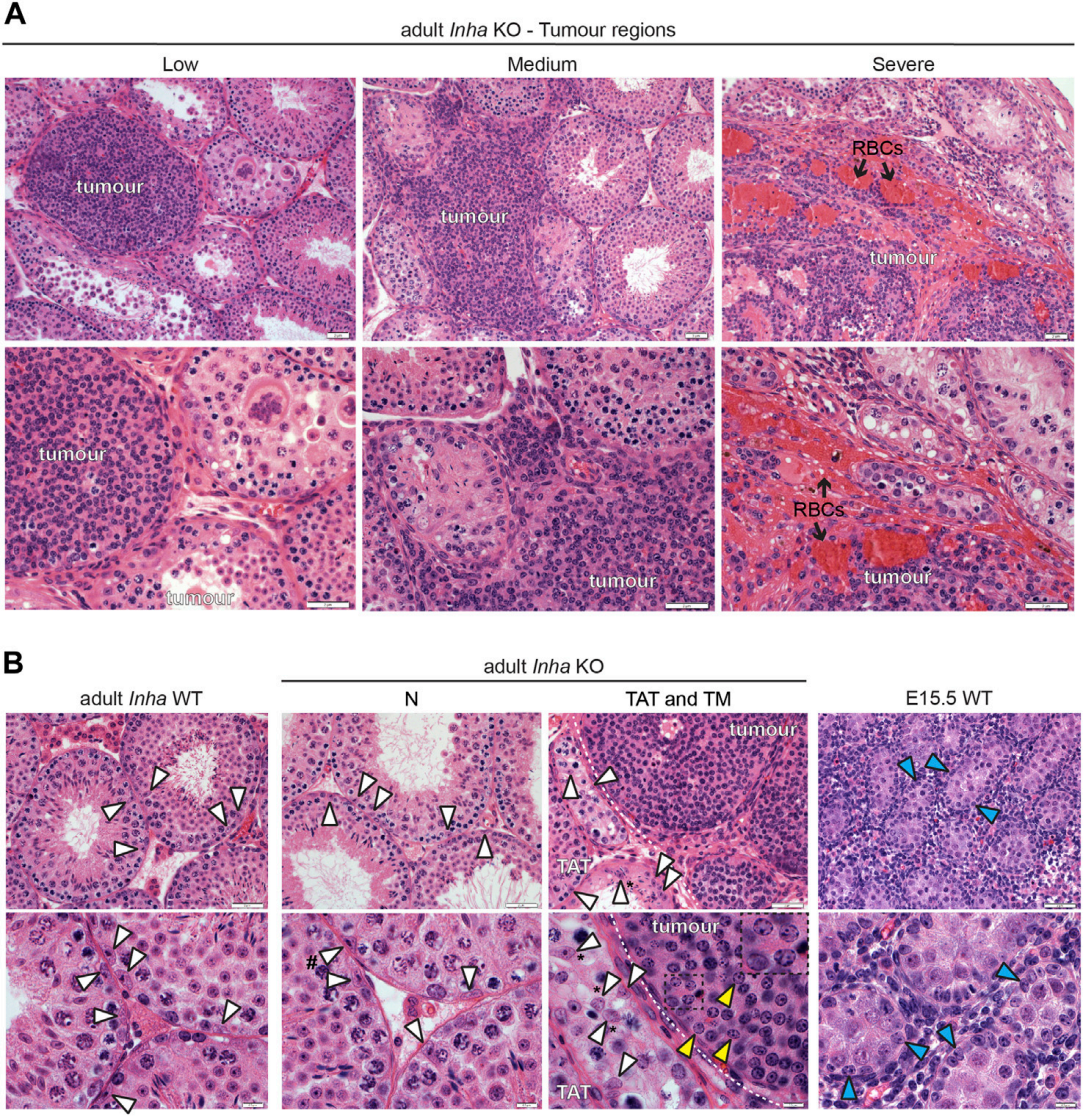
Histological features of tumours and Sertoli cells in *Inha* KO testes. **(A)** Representative H&E-stained images of low, medium, and severe grade tumours showing tightly packed tumour cells enclosed within a basement membrane (low), infiltration of tumour cells into interstitial areas (medium), and tumour lacking obvious tubules with red blood cells (RBCs, black arrow) (severe) in *Inha* KO testes. **(B)** A comparison of Sertoli cell nuclei in adult *Inha* WT, *Inha* KO N, TAT and tumour regions (TM) and fetal (E15.5 WT) testes. Sertoli cells nuclei exhibiting normal histological features (white arrowhead), tumour cells (yellow arrowhead and inset), and fetal Sertoli cells (blue arrowhead) are indicated. Sertoli cell nuclei displaced from basement membrane (indicated by *) and with tripartite nucleoli (indicated by #). Tumour border indicated by white dotted line. Scale bars = 0.5 µm (high magnification images, 7B, bottom panel) or 2 µm (all other images).

To investigate the cellular composition and transcriptional landscape of *Inha* KO tumours, we used 1) cell-specific transcripts to explore the relative proportion of testis cell types (germline, immune, Leydig, Sertoli, endothelial, and peritubular myoid cells) within the tumours (Figure 5), 2) immunofluorescence to examine the *in situ* distribution of these cell types (Figure 6), and 3) H&E staining to examine the cytological features and arrangement of cells within the tumours (Figure 7). Cell-specific transcripts, identified from single-cell RNAseq datasets of adult mouse testis (Hermann et al., 2018), allowed us to assess the representation of each cell type within the tumours. As expected, undifferentiated SPG markers (*Sall4*, *Plzf*) generally increased (mean CPM) in KO N, and TAT (Supplementary Table S2) consistent with our IF counting data. In the TM samples, these transcripts were elevated and highly variable. While this may reflect germ cell remnants present within these regions, SALL4 staining was low/absent, therefore the significance of these results in the context of the tumours is currently unclear. Germ cell markers (*Ddx4*, *Sycp3*) were significantly reduced in tumours, indicating the paucity of germ cells in these areas (Figure 5A). Immune cell markers (*Cx3cr1*, *Csf1r*) were increased in TAT and TM groups, consistent with immune cell infiltrates being a common feature (Figure 5B). Intriguingly, Leydig cell transcripts exhibited two distinct profiles; *Insl3* and *Klk1b21* were strongly decreased in all KO groups compared with WT, in accord with reduced Leydig cell number and/or function, however steroidogenic markers such as *Cyp11a1* and *Hsd17b3* were increased in TAT and TM groups indicating these regions display a steroidogenic phenotype (Figure 5C). Interestingly, certain Sertoli cell specific transcripts (*Sox9*, *Gata1*, *Rhox8*, *Cst12*) were increased in TAT, likely reflecting a proportional increase in Sertoli cells due to a decrease in germ cells but were not significantly different in TM regions compared to WT (Figure 5D). Endothelial (*Pecam1*, *Icam2*, *Kazald1*, *Ctla2a*) (Figure 5E) and peritubular myoid cell (*Acta2*, *Lsp1*, *Serpine1*, *Fbxl22*) (Figure 5F) transcripts were significantly increased in TAT and TM groups compared to WT. These likely reflect both the increased vascularisation, which is common in testicular germ cell tumours (TGCT) and tumours in general (Silvan et al., 2010; Ribatti and Pezzella, 2021), and an increase in the myoid cells that contribute to the seminiferous tubule basement membrane.

Next, we visualised the distribution of key cell types across the same tumour region using immunofluorescence (Figures 6A–D). Representative images show three tumours of different sizes and severity: low (L), medium (M) and severe (S) grade tumours (outlined white dotted lines). Tumours were classified based on the following criteria: visible tumour size together with the absence of normal spermatogenic architecture, specifically, the loss GCNA + germ cells and the presence of cell infiltrates. Although *Inha* KO tumours are thought to be derived from Sertoli cells, unexpectedly, SOX9+ cells, normally marking Sertoli nuclei, were less abundant in tumour regions and very low in the severe tumours despite being readily detected in *Inha* KO ‘normal’ and TAT tubules, (Figure 6A).

Although smaller tumours contained some germ cell remnants, more developed tumours did not contain germ cells (GCNA+). As outlined above, clusters of GFRA1+ cells were visible within the tumours and in tubules bordering the tumour regions (Figure 6A, arrowheads). SOX3, marking spermatogonial progenitors (Figure 6B, white arrows), was detected in *Inha* KO ‘normal’ tubules and also in tubules containing clusters of SSCs; these observations show these cells have the capacity to differentiate, albeit at a reduced rate. SPG and early meiotic germ cells (GILZ+) exhibited a similar staining pattern to GCNA and were not readily detected in the tumour regions (Figure 6B). The tubule basement membrane, outlined by LAMININ, appeared thicker in TAT and tumour regions than KO ‘normal’, consistent with our RNAseq data. In humans, the equivalent structure, the lamina propria, is thicker in azoo- and oligospermic patients (de Kretser et al., 1975; Sato et al., 2008), and in Leydig cell tumours (Charfi et al., 2012), providing an example of a feature common to these somatic cell tumours in mice and human testicular disorders.

The steroidogenic enzyme CYP11A1 exhibited remarkably intense staining in all tumours, including in low grade tumours, suggesting the steroidogenic phenotype is present early during tumour initiation (Figure 6C). Consistent with the formation of haemorrhagic tumours (Figure 4B) and our RNAseq data (Figure 5E), we observed increased levels and frequency of PECAM+ endothelial cells/vasculature around the tumour boundary and within severe grade tumours (Figure 6C). To support tumour expansion, blood vessel growth (angiogenesis) is induced via the secretion of various factors including FGFs and VEGF (Sakurai and Kudo, 2011), both of which were increased transcriptionally in the TM group (Figure 7B). Finally, immune cells were detected using the pan-leukocyte marker CD45. The frequency of elongated CD45^+^ cells observed was increased at the perimeter of seminiferous tubules close to the tumour boundary, around the tumour borders, and inside the tumours (Figure 6D, arrows) consistent with our RNAseq data showing *Ptprc* elevation.

Histological analyses on H&E-stained sections allowed us to examine the cellular and tissue structure of TAT and tumour regions in more detail and provided additional insights (Figures 7A,B). The classification of tumours which appear as low, medium, and severe grade (as described in Materials and Methods) provides clues to the events involved in tumour progression. In low and some medium severity tumours, tightly packed tumour cells were surrounded by a distinct border, likely the LAMININ+ basement membrane (observed in Figure 6B), providing evidence that the tumour originates from a cell/s within the tubule. As the tumour progresses, the border is less well-defined, and the tumour cells appear to infiltrate the interstitial space. In severe tumours, there is a lack of both tubules and basement membrane (Figure 6B, LAMININ staining), and the increased numbers of red blood cells (RBCs) is consistent with haemorrhagia (Figure 7A). Our interpretation based on these H&E images and LAMININ staining is that the tumours initiate within the tubule and proliferate to the extent that the tubule becomes enlarged, leading to eventual breakdown of the basement membrane. Subsequently the tumour cells infiltrate the local region causing progressive degeneration of surrounding tubules, loss of spermatogenesis, and increased vascularisation. Next, a histological comparison of tumour cells with Sertoli cells from adult *Inha* WT and KO tubules was performed (Figure 7B). Sertoli cell nuclei in *Inha* WT and KO N tubules (arrowhead, white fill) were identifiable based on their shape and position, parallel or perpendicular to the basement membrane. Depending on the plane of the section, characteristic nuclear indentations, and tripartite nucleoli were also visible. In *Inha* KO TAT tubules with incomplete spermatogenesis, Sertoli cell nuclei with a normal appearance were observed, however they often appeared displaced from the basement membrane. Nuclei within the tumour regions (arrowhead, yellow fill) did not display typical Sertoli cell characteristics, appearing rounded with chromatin granules arranged along the nuclear membrane (see inset), more closely resembling fetal Sertoli cells (arrowhead, blue fill) (Figure 7B).

Overall, our RNAseq, immunofluorescence and H&E data were in accord: tumour progression results in a loss of germ cells and mature Sertoli cells, accompanied by an increase in the proportion of peritubular myoid cells, vascular endothelial cells and immune cells. Intriguingly, tumour cell nuclei more closely resemble fetal compared to adult Sertoli cell nuclei and exhibit evidence of an increasingly altered steroidogenic profile with tumour progression.

### *Inha* KO tumours express multiple growth factors associated with SSC maintenance

**FIGURE 8:**
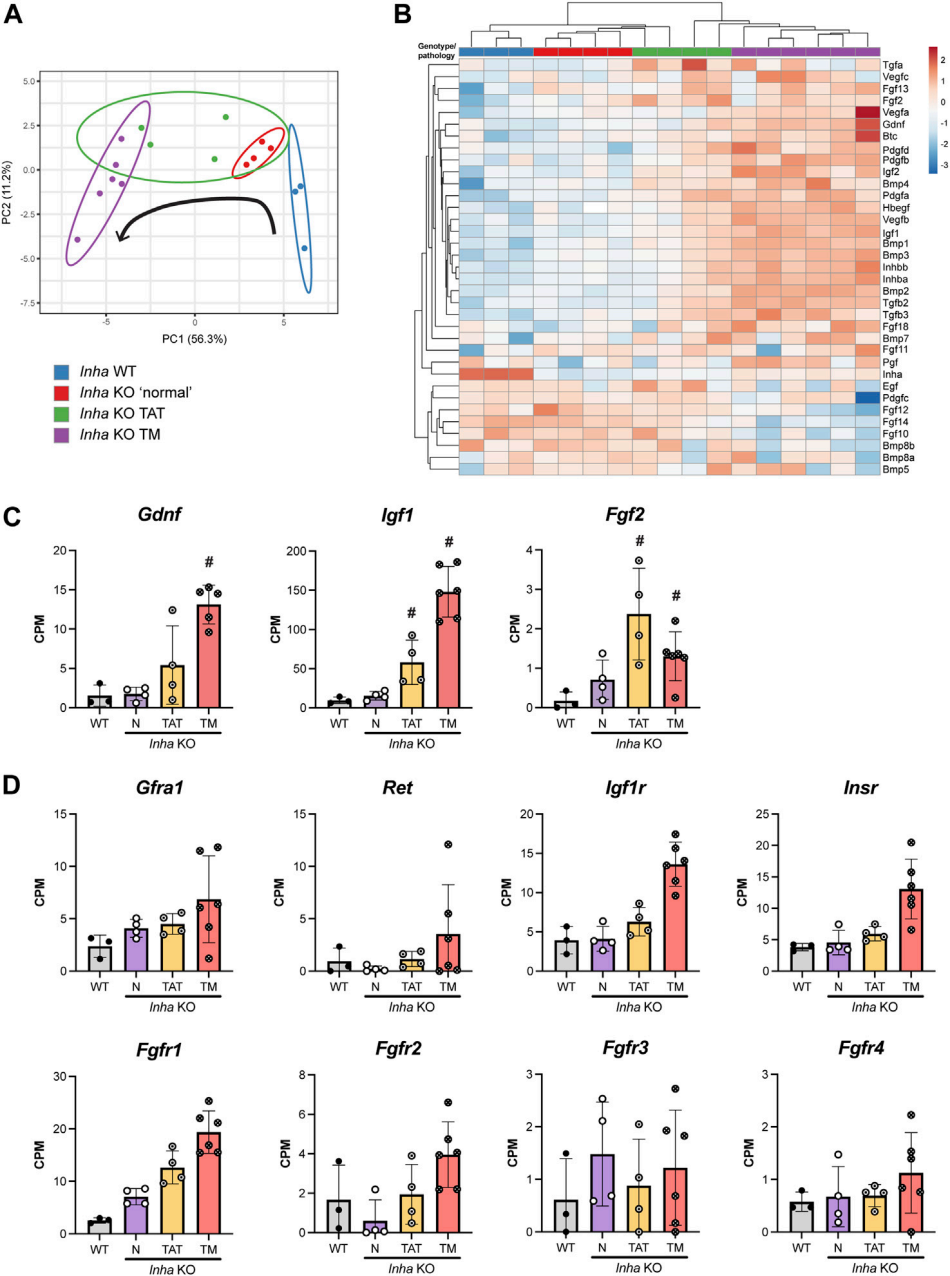
Profile of growth factor expression in adult *Inha* testes. **(A)** PCA plot shows growth factor expression diverges from *Inha* WT to KO TM (direction indicated by black arrow). **(B)** Heatmap showing 35 growth factors measured in *Inha* WT and KO samples. Both rows and columns are clustered using correlation distance and average linkage. **(C)** Growth factor transcripts associated with maintenance of SSCs (*Gdnf*, *Igf1*, *Fgf2*) and **(D)** associated receptors are increased in TAT and TM regions. All graphs show Counts Per Million (CPM) from RNAseq analysis of *Inha* WT tubules (grey) and *Inha* KO ‘normal’ N (purple), *Inha* KO ‘tumour associated tubules’ TAT (orange), *Inha* KO ‘tumours’ TM (red) (n = 3–6). Mean ± SD. Significance (indicated by #) determined in DEGUST (FDR <0.05, Abs logFC >0.05).

Within seminiferous tubules, SSCs respond to exogenous growth factors that regulate their self-renewal and differentiation. One example is GDNF, secreted by Sertoli cells in a cyclic manner to regulate spermatogonial stem cell proliferation (Meng et al., 2000; Sharma and Braun, 2018). The observation of GFRA1+ SPG clusters in tubules adjacent to tumours lead us to hypothesise that *Inha* KO tumours secrete factors that promote SSC self-renewal and maintenance. To corroborate this, and potentially identify new factors that affect signalling important to SSC biology, we profiled growth factor transcript expression in *Inha* WT and KO testes. Our initial analyses comprised 53 growth factors including TGFβs, BMPs, FGFs, IGFs, PDGFs, and VEGFs, and following exclusion of those below the level of detection, 35 growth factors remained. The PCA plot showed growth factor transcript expression progressively diverges from *Inha* WT to KO TM (Figure 8A, black arrow). The heatmap revealed a suite of growth factor transcripts that increase from WT tubules to *Inha* KO tumours (Figure 8B), including *Inhba* (activin A), consistent with previously published data showing activin A levels are elevated in these mice (Matzuk et al., 1994). We found *Gdnf*, *Igf1* and *Fgf2* transcripts, involved in maintaining SSCs in an undifferentiated state (Meng et al., 2000; Kubota et al., 2004; Ishii et al., 2012; Sharma and Braun, 2018), were significantly increased in TAT and/or TM regions (Figure 8C), as were *Bmp4* and *Inhba* which promote SSC differentiation *in vitro* (Nagano et al., 2003). Given the increased numbers of GFRA1+ SPGs observed in our model, and the elevation of transcripts important for both SSC self-renewal and differentiation, we surmise that the relative stoichiometry of growth factors is critical in determining cell fate outcomes.

Importantly for SSC biology, secretion of FGFs (4, 5 and 8) by lymphatic endothelial (LE) cells located near the vasculature, regulate SSC density and fine tune their self-renewal and differentiation in normal mouse testes (Kitadate et al., 2019). However, transcripts encoding *Fgf4*, *5* and *8* (plus others) were below the level of detection in all samples. Of the remaining FGF transcripts, 7 were detected, and their expression was variable in the *Inha* KO TAT and TM regions; we measured increased levels of *Fgf2*, *11*, *13*, and *18* transcripts, and decreased levels of *Fgf10*, *12* and *14* compared to WT (Figure 8B). *Fgf2* and *Fgf18* encode secreted canonical FGFs, which bind to and activate FGFRs, however *Fgf11* and *13* transcripts encode non-signalling, intracellular proteins (Ornitz and Itoh, 2015). In testes, FGF2 is a key factor in promoting SSC expansion *in vitro*, and a lack of FGF2 leads to dysregulated sperm production and altered sperm morphology and function (Saucedo et al., 2018), while relatively little is known about the function of FGF18. Since *Fgf10*, *12* and *14* are predominantly expressed in germ cells (Hermann et al., 2018), their decrease likely reflects the loss of germ cells in these regions.

Receptors related to GDNF, IGF and FGF signalling were detectable and generally increased in the TAT and TM samples signifying the potential for these signalling pathways to be active (Figure 8D). Downstream targets of FGF (*Stat1*, *3*, *5*, *Foxo1*, *Etv4*), GDNF (*Bcl6b, Egr2, Egr3*) and IGF (*Igf1*, *Trp53*, *Casp8*) signalling were increased in tumours, indicating pathway activation (data not shown). GDNF receptor transcripts *Gfra1* and *Ret* were present at low levels, and *Gfra1* transcripts were increased in N and TAT samples compared to WT, consistent with our counting data. Both IGF1 receptors, *Igf1r* and *Insr* were detected and FGF2 receptors, *Fgfr1* - *Fgfr4*, exhibited varying expression levels. *Fgfr1* was most highly expressed and increased uniformly in relation to the tumour proximity, however *Fgfr2* - *4* were expressed at low levels and were highly variable. These data indicate that progression towards a tumour phenotype results in increased production of numerous growth factors including several of known importance in SSC maintenance, some of which are not exclusive to a particular cell type.

## Discussion

In studying the importance of activin A to testis biology, we and others have previously investigated how inappropriate levels of activin A affect normal spermatogenesis (Mendis et al., 2011; Whiley et al., 2020). Tight regulation of its action and impact on a wide variety of processes relating to testis biology have been documented (Boitani et al., 1995; Barakat et al., 2008; Itman et al., 2009; Mendis et al., 2011; Young et al., 2015; Wijayarathna and de Kretser, 2016). In culture, activin A promotes differentiation of mouse (Nagano et al., 2003) and human SPG (Tan et al., 2020a), however its direct impact on spermatogenesis in the adult testis *in vivo* is unknown. To address this, we examined *Inha* KO mice which have elevated activin A bioactivity and develop stromal tumours in adulthood. The surprising observation of discretely localised clusters of SPG close to the tumours led us to investigate how these could arise and be sustained. Tubules distant from tumour foci exhibit grossly ‘normal’ spermatogenesis, however spermatogenic deterioration was evident in tubules adjacent to tumours, compelling us to consider these regions independently. This approach led us to use the *Inha* KO mice as a model with which to interrogate the bioavailability of niche growth factors that influence germline fate.

### Increased SSC and proposed models

Our analyses showed the ‘normal’ *Inha* KO testis tubules contain a higher proportion of undiff SPG and an expanded pool of GFRA1+ SPGs (∼2-fold higher), indicating increased SSC abundance. In addition, both cell types were more proliferative than in WT controls. The increase in GFRA1+ cells was surprising given that *in vitro* studies have demonstrated SPG treated with a 50 ng/mL activin A promotes their differentiation (Nagano et al., 2003)*.* Although activin A serum (Matzuk et al., 1994) and transcripts (our data) are elevated in adult (4–6 week old) *Inha* KO testes, it is highly likely that within the complex *in vivo* cellular environment, these levels may not be robust enough to elicit differentiation of SPG. Indeed, it is possible that in culture 50 ng/mL activin A represents a supraphysiological level that far exceeds the elevated levels of activin A in even the *Inha* KO mice. Clusters of primitive SPG and a decreased proportion of progenitors in tubules adjacent to tumour regions indicated that the tumour cells provide a localised microenvironment that supports SSC self-renewal and impedes their differentiation. In agreement, transcriptional analyses demonstrated tumour areas were enriched for transcripts encoding numerous growth factors including several fundamental to SSC self-renewal (*Gdnf* and *Fgf2*). In total, over 20 additional growth factors transcripts with the potential to influence SSC biology were also increased, including those shown to promote SSC differentiation *in vitro* (*Inhba*, *Bmp4*) (Nagano et al., 2003; Li et al., 2014). Because SSCs reside within a complex changing cellular environment and are exposed to numerous growth factors simultaneously, the relative stoichiometry of growth factors may be critical to determining stem cell fate. For example, can we identify a concentration at which GDNF overrides BMP4 differentiation cues in SSCs?

**FIGURE 9:**
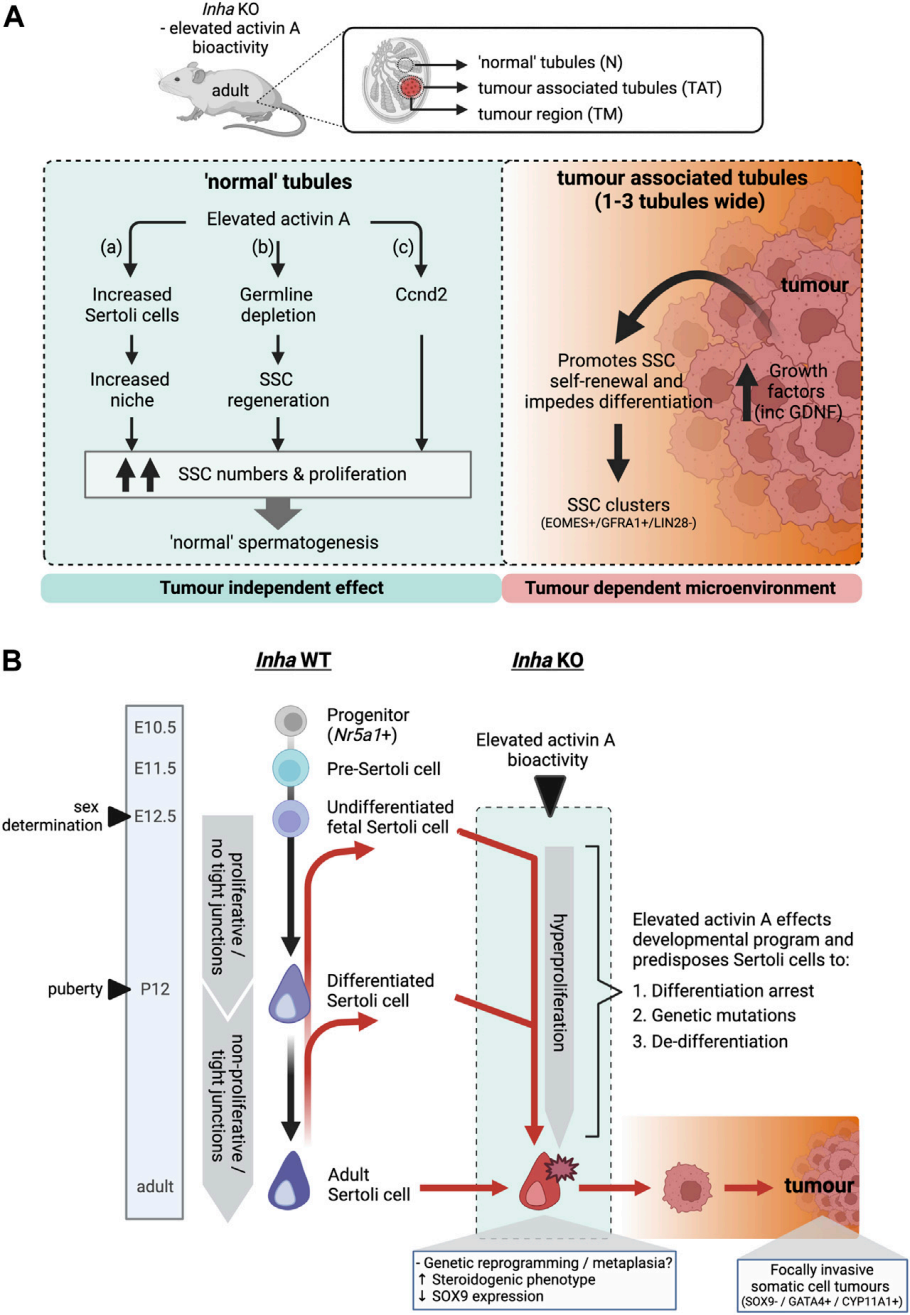
**(A)** Schematic outlining the effects of elevated activin A bioactivity on spermatogonial fate in *Inha* KO mice. Proposed model of action in ‘normal’ tubules (left) and tumour associated/tumour regions (right). In ‘normal’ tubules without evident tumours, elevated activin A bioactivity increases SSC numbers and proliferation, via (a) increased Sertoli cells and therefore increased SSC niche availability, (b) germline depletion event during early post-natal life leads to subsequent SSC regeneration, or (c) regulating *Ccnd2* expression, resulting in grossly ‘normal’ spermatogenesis. In contrast, *Inha* KO tumours produce increased levels of numerous growth factors including GDNF. This creates a microenvironment that promotes SSC self-renewal and impedes differentiation in tumour associated tubules, resulting in clusters of SSCs. **(B)** A proposed model of the origin of *Inha* KO somatic cell tumours. In *Inha* WT animals, Sertoli cells originate from an *Nr5a1*+ progenitor population, transition into pre-Sertoli cells (E11.5) and undifferentiated fetal Sertoli cells at the time of sex determination (E12.5). Sertoli cells remain undifferentiated (proliferative and lacking tight junctions) until puberty (P12). After puberty, Sertoli cells become differentiated, form tight junctions and are predominantly non-proliferative (black arrows). In *Inha* KO testes, activin A transcripts increase directly after sex determination at E12.5, resulting in hyperproliferation of Sertoli cells throughout testis development. We speculate this environment alters the developmental program of Sertoli cell (red arrows) resulting in 1. differentiation arrest of a progenitor population at stage unknown, 2. acquisition of genetic mutations and/or 3. de-differentiation to a less mature state. Genetic reprogramming results in the acquisition of a steroidogenic phenotype together with loss of SOX9 expression early during tumour progression. Created with BioRender.com.

Based on histological features such as SSC clusters, spermatogenic deterioration, and increased transcriptional dysregulation in tubules bordering tumour regions, we propose that tumour-derived growth factors influence a localised microenvironment ∼1-3 tubules wide surrounding the tumours. Therefore, the increased numbers of SSC within ‘normal’ tubules, distant from the tumours was surprising. Below we discuss three potential tumour independent mechanisms of actions based on our findings that could result in the increased frequency of SSCs within *Inha* KO ‘normal’ tubules (summarised in Figure 9A) and speculate that one or more of these contributes to the observed phenotype.

In the first model (a), Sertoli cell niche-driven: The roles of activin A on Sertoli cell proliferation and maturation throughout testis development are well documented (Boitani et al., 1995; Mendis et al., 2011; Nicholls et al., 2012; Itman et al., 2015; Haverfield et al., 2017). In *Inha* KO testes, Sertoli cell numbers are higher than in WT (Haverfield et al., 2017), and adult *Inha* HET testes support an increased (44%) daily sperm production (Itman et al., 2015). Because each Sertoli cell supports a fixed number of germ cells, and their postnatal complement determines SSC numbers in adult mice (Orth et al., 1988; Oatley et al., 2011), we considered the relationship between the activin driven increase in Sertoli cell number and GFRA1+ SPGs. As expected, both SOX9+ Sertoli and GFRA1+ cells were elevated in *Inha* KO testes, however the GFRA1+/SOX9+ cell ratio (Supplementary Figure S3A) remained significantly higher (∼50%) in *Inha* KO testes compared to WT, indicating that Sertoli cell numbers do not solely contribute to SSC numbers in this model. The increased Sertoli cell population likely aids in supporting the expanded stem cell pool.

In the second model (b), Germ cell catchup/regeneration: Germ cell depleted tubules observed in P16 *Inha* HET testes were resolved by P28 (Itman et al., 2015), indicating that germ cell loss due to increased activin A bioactivity in developing testes induces a germline regenerative process. Our unpublished observations indicate that germ cell numbers are reduced by approx. 50% in neonatal *Inha* KO mouse testes compared to controls. In adult *Inha* KO ‘normal’ tubules with ongoing spermatogenesis, the full complement of germ cells is visible. We therefore propose establishment of SSCs in juvenile life set the size of the stem cell pool for adult life and there is an ongoing regenerative response by SSCs. In damaged testicular tissue following busulfan-induced germline depletion, SSCs exhibit molecular characteristics different from homeostatic SSCs, including increased expression of cell cycle genes, and activation of the PI3K/AKT and mTORC1 signalling pathways (La et al., 2018a; Kitadate et al., 2019; La et al., 2022). The undifferentiated spermatogonia in *Inha* KO testes were more frequently proliferative (Ki67+), and cell cycle transcripts (*Ccnd2*, *Mki67*, *Kif4, Top2a*, *Smarca5*, *Hells*) were elevated, consistent with a regenerative SSC phenotype (La et al., 2022) (Supplementary Table S2). In contrast, as a readout of P13K/AKT activity, we found FOXO1 was nuclear in SPG of *Inha* KO N and TAT tubules, indicative of a homeostatic environment ((Goertz et al., 2011; La et al., 2022); Supplementary Figure S3B). Additionally, transcripts encoding several regenerative SSC markers were undetectable (e.g., *Eomes*, *Pax7*, *Plaur*) (Sharma et al., 2019; La et al., 2022), and EOMES^+^ cells were decreased as a proportion of the PLZF + population, compared to in *Inha* WT testes. Thus, SSCs in *Inha* KO ‘normal’ tubules exhibit some features of regenerative SSC but lack others.

In the third model (c), activin A-driven: CCND2 (Cyclin D2) acts synergistically with CCNE (Cyclin E) to induce SSC proliferation without the requirement for self-renewal factors such as GDNF and FGF (Lee et al., 2009). The cell cycle transcript *Ccnd2* was 1 of only 9 transcripts elevated in *Inha* KO ‘normal’ tubules (Figure 4F; Supplementary Figure S2) and is activin A regulated in several tissues, including in embryonic mouse testes lacking activin A (*Inhba* KO) (Park et al., 2005; Mendis et al., 2011; Pauklin and Vallier, 2013). By sc-RNAseq, *Ccnd2* is predominantly expressed in undiff SPG, but it is also produced in endothelial, Leydig and peritubular myoid cells in adult testes (Hermann et al., 2018), therefore we propose activin A may act as a niche factor, promoting SSC self-renewal via Cyclin D2*.* Intriguingly, transplantation of SSCs overexpressing both CCND2 and CCNE1 produced tubules consisting of spermatogonial clusters and an absence of haploid cells, resembling germ cell tumours (Lee et al., 2009) and similar to TAT tubules. In contrast, *in vitro* studies have shown activin A promotes SSC differentiation (Nagano et al., 2003; Tan et al., 2020a). Nevertheless, our *in vivo* results suggest that elevated activin A levels may contribute to increased SSC renewal in *Inha* KO ‘normal’ tubules via upregulation of *Ccnd2*.

### Tumour landscape offers clues to developmental origins

Tumour cells can originate from progenitor cells that fail to differentiate appropriately (i.e., testicular germ cell tumours (TGCT) (Rajpert-De Meyts, 2006)), or they may arise via reversion to a more immature state. *Inha* KO male mice develop focally invasive gonadal sex cord-stromal tumours, thought to originate from Sertoli cells. Because these tumours initiate from foci, we considered that elevated activin A levels in fetal and postnatal life sustains or modifies a precursor somatic cell population, preserving it in an inappropriate developmental state that manifests as a tumour in adulthood (Figure 9B). Indeed, the presence of cell nuclei resembling fetal Sertoli cell nuclei surrounded by an intact basement membrane in low grade tumours suggests the tumour mass is derived from cells initially present within the tubules (i.e., Sertoli or germ cells). To determine if these tumours possess a signature consistent with immature Sertoli cells, we searched for Sertoli cell-specific transcripts selectively expressed in either undifferentiated or differentiated Sertoli cells based on sc-RNAseq data (Hermann et al., 2018)) and prior knowledge (Haverfield et al., 2015). *Amh*, marking immature Sertoli cells, was not detectable in our RNAseq dataset, while levels of mature Sertoli cells markers, *Gata1* and *Cldn11*, were not different compared to WT, indicating the tumour cells had not acquired an immature Sertoli cell signature. These findings are limited by the small number of genes fitting the criteria, and lineage tracing studies and/or single-cell RNAseq analyses would address this.

The combination of transcriptional and histological analyses provided new insights into *Inha* KO tumour formation and their molecular and cellular characteristics. Small, low-grade tumours are initially surrounded by an intact basement membrane, while larger more ‘advanced’ tumours appear to invade the interstitial space, engulfing nearby tubules, such that remnants of intact tubules were frequently observed within severe tumour regions. This may occur due to tubule basement membrane breakdown, angiogenesis and hyperproliferation/infiltration of tumour cells. There is a signature consistent with elevated levels of several cell types (immune, endothelial, and peritubular myoid cells) and the deficiency in germ cells manifests as spermatogenic failure in these tumours.

Interestingly, *Inha* KO tumours exhibited changes to hallmark proteins and transcripts associated with Sertoli and adult Leydig cells indicative of cell identity changes that can occur within a tumour environment. Although the tumours were GATA4+ (Haverfield et al., 2017), Sertoli cell-specific transcripts were not increased (Figure 5D) and SOX9 protein, a marker of both immature and mature Sertoli cells, was not detected (Sharpe et al., 2003). As *Sox9* transcripts were not altered compared to WT, this suggested post-transcriptional and/or post-translational mechanisms may limit SOX9 levels in these tumours. SUMOylation (small ubiquitin-like modifier) is a key mechanism by which SOX9 proteins are repressed (Williams et al., 2020), and we measured elevated *Sumo2* and *Sumo3* transcript levels in TAT and TM regions, suggesting this is one mechanism by which SOX9 protein expression may be disrupted (Supplementary Figure S2). Whether the loss of SOX9 protein is a signature of tumorigenic Sertoli cells remains to be elucidated. Additionally, transcripts encoding steroidogenic enzymes (*Cyp11a1*, *Hsd3b1*, *Cyp17a1*, *Hsd17b1, Hsd17b3, Cyp19a1*) and protein (CYP11A1) found in adult Leydig cells were elevated in both *Inha* KO TAT and tumour regions when compared to *Inha* KO ‘normal’ tubules, leading us to suggest cells in these regions acquire an atypical steroidogenic phenotype. Metaplasia refers to the replacement of one differentiated somatic cell type with another, a response typical of chronic exposure of cells to an irritant, and a known precursor to certain cancers (Giroux and Rustgi, 2017). In *Inha* testis tumour regions, the combination of low SOX9 protein levels and increased expression of steroidogenic components allude to cell identity changes consistent with this process (Figure 9B).

In adult testes, appropriate steroid production relies on a functional Leydig cell population. Adult *Inha* KO mice have reduced Leydig cell numbers (Matzuk et al., 1992) (Figure 7B; Supplementary Figure S3C); this is supported by RNAseq data showing an approximately 5-fold reduction in Leydig cell transcripts (*Insl3*, *Klk1b21*) in *Inha* KO ‘normal’ tubules, TAT tubules and tumours. Steroidogenic enzyme transcripts and protein (listed above) were elevated in *Inha* KO TAT tubules and tumours compared to *Inha* KO ‘normal’ tubules and IPA analyses identified lipid metabolism to be altered in *Inha* KO testes (N, TAT and TM samples, Supplementary Table S2). The observation that tubules are enriched for steroidogenic markers as they transition towards a tumorigenic phenotype, regardless of tumour size and severity, was intriguing. The enzymes CYP11A1, HSD3B1, and HSD17B1/B3 catalyse conversion of cholesterol to testosterone, while CYP19A1 catalyses androstenedione and testosterone to estrone and estradiol respectively, providing considerable scope for dysregulated steroid production. How reduced Leydig cell numbers, together with altered expression of steroidogenic enzymes impacts on the steroid milieu in *Inha* KO adult testes is currently under investigation.

The origin of these tumours remains open to further study. It is known that Sertoli cell development and proliferation are regulated by several hormones including activin A and FSH, (reviewed in (Meroni et al., 2019)), both of which exhibit elevated serum levels in adult *Inha* KO mice (Matzuk et al., 1992). Consistent with a role in driving Sertoli cell proliferation and tumour formation, disruption of luteinising hormone (LH) and/or FSH signalling ablates or reduces tumour size (Kumar et al., 1996; Haverfield et al., 2017). Our histological data showing an accumulation of tumour cells within low severity tumour tubules suggests the tumours are likely derived from Sertoli cells.

In summary, the data presented here show the condition of chronically elevated activin A can modify the adult testis SSC pool. *Inha* KO somatic cell tumours lack SOX9, a key hallmark feature of adult Sertoli cells and exhibit an unexpected steroidogenic profile, while providing a microenvironment that supports SSC self-renewal. Our identification that these tumours produce growth factors of potential importance to SSC biology highlight its value for studies of SSC regulation. Further examination of this model may help explain the aetiology of testis stromal cell tumours (Ulbright et al., 2000), and we propose that cells derived from tumours may be employed as feeders to help improve *in vitro* SSC culture that can be relevant for therapeutic purposes (Tran et al., 2022).

## Data Availability

The data presented in this study are deposited in the NCBI GEO repository, accession number GSE236488.
