# Supplementary figures and images for: Spermatogonial fate in mice with increased activin A bioactivity and testicular somatic cell tumours
Source: Front Cell Dev Biol. 2023 Jul 26;11:1237273. doi: 10.3389/fcell.2023.1237273 (PMC10409995; doi:10.3389/fcell.2023.1237273)

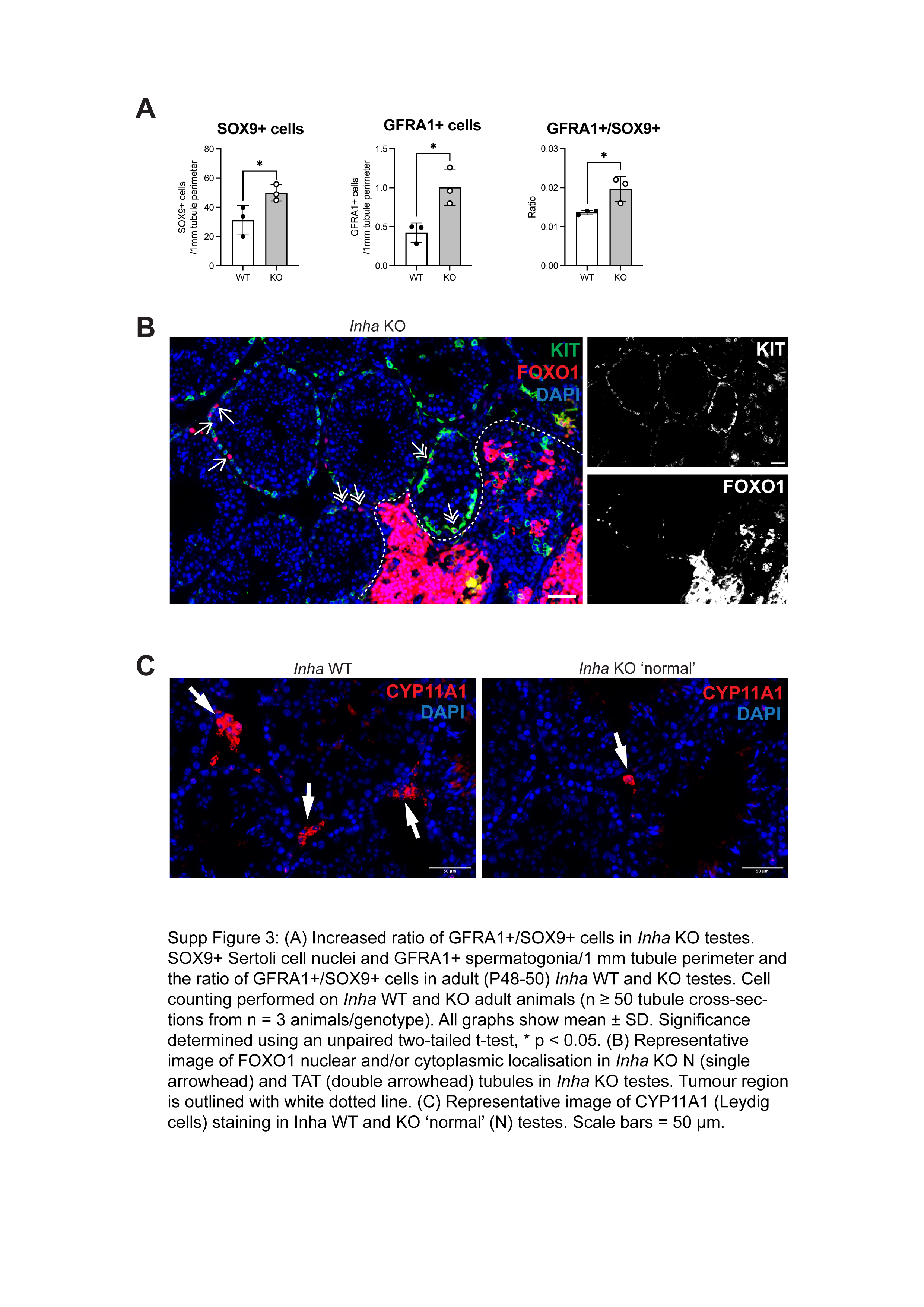

Supplement: Supplementary file 2 [file Image3.tif]

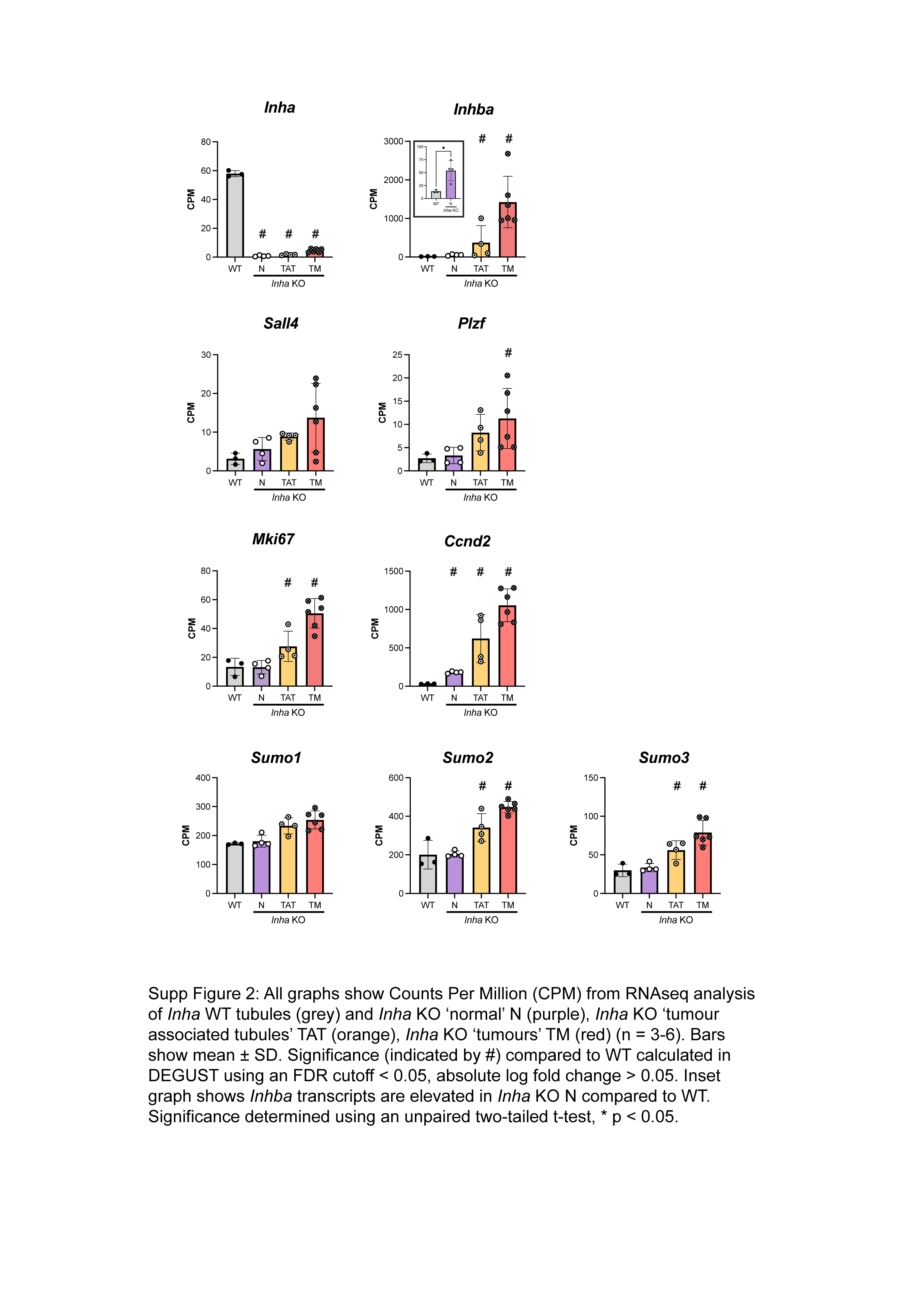

Supplement: Supplementary file 3 [file Image2.tif]

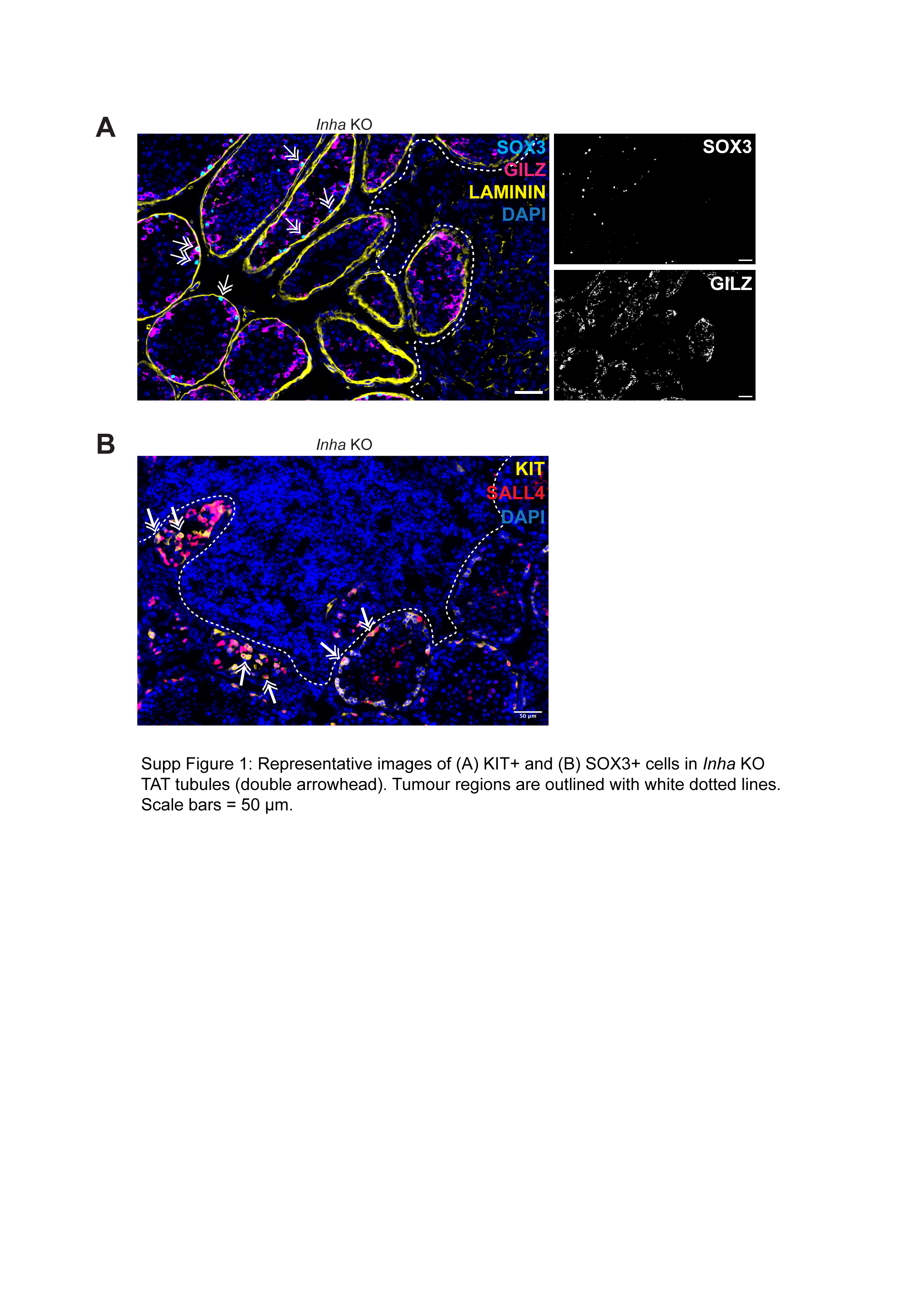

Supplement: Supplementary file 4 [file Image1.tif]
